# Effect of acupuncture and moxibustion on the immune function of patients with malignant tumors: a systematic review and meta-analysis

**DOI:** 10.3389/fimmu.2025.1583522

**Published:** 2025-07-25

**Authors:** Yan Wang, Bailu Sui, Ying Zhang, Liyuan Fang, Yi Xie, Yuhang Fang, Runxi Wang

**Affiliations:** ^1^ Department of Oncology, Guang’anmen Hospital, China Academy of Chinese Medical Sciences, Beijing, China; ^2^ Graduate School, Beijing University of Chinese Medicine, Beijing, China

**Keywords:** acupuncture, moxibustion, T lymphocyte cells, malignant tumors, meta-analysis

## Abstract

**Background:**

Acupuncture and moxibustion have been shown to be safe and effective methods for bidirectional immunomodulatory function. Clinical practice and many studies have shown that acupuncture and moxibustion have a certain clinical effect on immune promotion in patients with malignant tumors.

**Methods:**

Eight electronic databases were searched systematically for articles published through December 31, 2024. Study Selection Randomized controlled trial studies (RCTs) that reported The number of T lymphocytes cells in patients with malignant tumors who received acupuncture and/or moxibustionon treatment were included. For continuous variables, effect estimates were calculated as mean difference (MD); and for dichotomous variables, the risk ratio (RR) was calculated. A funnel plot was used to analyze potential publication bias.

**Results:**

33 studies involving 2610 participants were included. Patients who received acupuncture and/or moxibustion treatment had higher CD3^+^, CD4^+^, CD4^+^/CD8^+^ and natural killer (NK) cell levels, but lower CD8^+^ levels. At the same time, the anti-tumor treatment effect was better than that of the control group.

**Conclusions:**

Evidence from this meta-analysis, acupuncture and moxibustion can enhance the immune function and improve the prognosis of malignant tumor patients. Further studies are recommended to support and confirm these findings.

**Systematic Review Registration:**

https://www.crd.york.ac.uk/prospero/, identifier CRD42023465759.

## Introduction

According to Global Cancer Statistics 2020, there are 19.3 million new cancer cases and nearly 10 million cancer deaths estimatedly in worldwide, while the incidence and mortality of cancer are increasing year by year. It is estimated that by 2040, the global cancer burden will reach 28.4 million cases, an increase of 47% over 2020. The global situation of cancer prevention and control is grim (1). A weakened immune system leads to the development of tumors, and studies have shown that immune function is closely related to the prognosis of cancer (2). Recently, immunotherapy based on activating and enhancing the patient’s immune system has shown anti-tumor effects (3). Immunotherapy mainly includes immune checkpoint inhibitors, tumor antigen vaccines, and immune stimulating cytokines (4). These treatment methods can reshape the tumor microenvironment, enhance the immune function, strengthen anti-tumor immune responses, and thus suppress tumor growth and recurrence (5).

However, there are still some limitations and challenges in the application of immunotherapy in cancer at present (6). For example, the effectiveness and sustainability of immunotherapy are still not ideal, and individual differences are large. Some patients have poor tolerance to immunotherapy (7). In addition, some patients may also experience immune-related adverse reactions during immunotherapy, which may affect the treatment effect (8).

Acupuncture and moxibustion demonstrate bidirectional immune modulation (9). In cancer, these therapies synergistically boost anti-tumor immunity through distinct mechanisms. Electroacupuncture enhances lymphocyte populations and granzyme B secretion while activating interferon-mediated signaling pathways (10). Moxibustion suppresses adrenaline-driven signaling to activate natural killer (NK) cell activity and limit tumor growth, while also reducing regulatory T cell (Treg) infiltration in the tumor microenvironment—a strategy that curtails immune evasion and tumor progression (11, 12). Clinical observations further support their role in improving cancer patients’ immune function and prognosis, such as acupuncture at ST36 increasing NK cell counts and reducing tumor burden in cervical cancer (13, 14). Together, these findings underscore the capacity of acupuncture and moxibustion to reprogram immune responses, tipping the balance toward anti-tumor surveillance while mitigating immune dysregulation.

There is still a lack of systematic reviews of the effect of acupuncture and moxibustion on the immunity of patients with malignant tumors. This study aims to demonstrate and quantify the effect of acupuncture and moxibustion on the immune function of patients with malignant tumors, especially the number of T lymphocyte subsets, and to analyze the improvement of prognosis and quality of life of patients with malignant tumors.

## Methods

This systematic review and meta-analysis was performed according to the guideline of Preferred Reporting Items for Systematic review and Meta-Analysis Protocols (PRISMA-P) 2015 (15). Procedures and study inclusion criteria were registered in PROSPERO (CRD42023465759) (https://www.crd.york.ac.uk/prospero/).

### Data sources and search strategy

A systematic search for articles published in electronic databases (PubMed, Cochrane Library, Embase, Web of Science, China National Knowledge Infrastructure, Wanfang database, VIP database and Chinese BioMedical Literature database) through December 31, 2024, was performed with no language or time restrictions. Medical subject headings (MeSH) terms and free text terms were used to obtain more comprehensive studies. The MeSH terms of “Acupuncture”, “Electroacupuncture”, “Moxibustion”, “Neoplasms”, “cancer”, “Immunity”, “T-Lymphocytes” were used to construct search Electroneedle, fire needle.

### Eligible criteria

#### Inclusion criteria

1. The patients diagnosed with solid malignant tumor by histopathology;
2. Only RCTs were included;
3. The control group received conventional therapies (e.g., chemoradiotherapy, radiotherapy, surgical treatment, traditional Chinese Medicine (TCM) treatment), and intervention groups received what control groups received plus acupuncture (including electroacupuncture, fire needle et.al) and/or moxibustion treatment;
4. The outcomes included immune function index (e.g., CD3^+^, CD4^+^, CD8^+^ or CD4^+^/CD8^+^).

#### Exclusion criteria

1. Repeated publication;
2. Outcome of interest not included;
3. Original data cannot be obtained by contacting the original author.

### Study selection and data extraction

EndNote 21 was used to manage literature. Two researchers (YW and BLS) independently retrieved the titles and abstracts of all articles. Any disagreement in screening process should be consulted with another researchers (YZ) to make a decision. The relevant information were independently extracted and cross-checked by two researcher (YW and BLS) independently, which including: 1) basic information of the article: author’s name, year of publication, study type, and sample size; 2) patient characteristics: age, gender, cancer typology, pathological types and disease stage; and 3) treatment outcomes: clinical intervention, main points of acupuncture and/or moxibustionon, number of intervention, duration of intervention, and outcomes. Disagreements were solved by discussion or consulting third-party opinion (YZ). Imputing a change-from-baseline standard deviation (SD) and mean using a correlation coefficient. A SD of the change from baseline for the experimental intervention was input, using following formula:


$$\begin{array}{c} SD_{E,\;change}=\;\surd [SD_{E,\;baseline}^{2}+\;SD_{E,\;final}^{2}-\;(2\;\times \;Corr\;\\ \times \;SD_{E,\;baseline}\times \;SD_{E,\;final})];\;Corr=0.75 \end{array}$$

(16)

Mean value of the change from baseline for the experimental intervention was input, using:


$$Mean_{E,\;change}=\;Mean_{E,\;final}-\;Mean_{E,\;baseline}$$

(17)

All data were rounded to two decimal places.

### Outcomes of interest

The primary outcome of this study was immune function, mainly evaluated with the number of T lymphocyte subsets, including CD3^+^, CD4^+^, CD8^+^ or CD4^+^/CD8^+^. The secondary outcomes included the number of NK cell and clinical effective rate. The clinical effective rate = complete response (CR) + partial response(PR). CR and PR were defined by Response Evaluation Criteria in Solid Tumors (RECIST).

### Assessment of study quality

Two researchers (YW and BLS) used the Cochrane Risk of Bias Tool (RoB) (18) to evaluate the methodological quality of all included RCTs independently. The following seven domains were assessed: random sequence generation, allocation concealment, blinding of participants and personnel, blinding of outcome assessment, incomplete outcome data, selective reporting and other biases. The included RCTs were assessed as low, uncertain, or high risk of bias. The results were shown in RoB graph.

### Statistical analysis

Review Manager software (version 5.4.1) was used to perform the meta-analysis. The random-effect model were used to synthesize evidence. Sensitivity or subgroup analysis were conducted to determine the cause of heterogeneity if it exists. The method of deleting studies one by one needed to be used to perform sensitivity analysis of the results to ensure stability. The subgroup analysis of the meta-analysis results for each outcome was required. The subgroup only includes items related to the comparison. Subgroup analysis was performed based on cancer typology and clinical interventions. For continuous variables, effect estimates were calculated as mean difference (MD); and for dichotomous variables, risk ratio (RR) were calculated. The effect estimates with their 95% confidence intervals (CI) were presented in the forest plots. If meta-analysis was not suitable, descriptive analysis was performed. Funnel plot was used to analyze potential publication bias. P < 0.05 was considered statistically significant.

## Results

**Figure 1:**
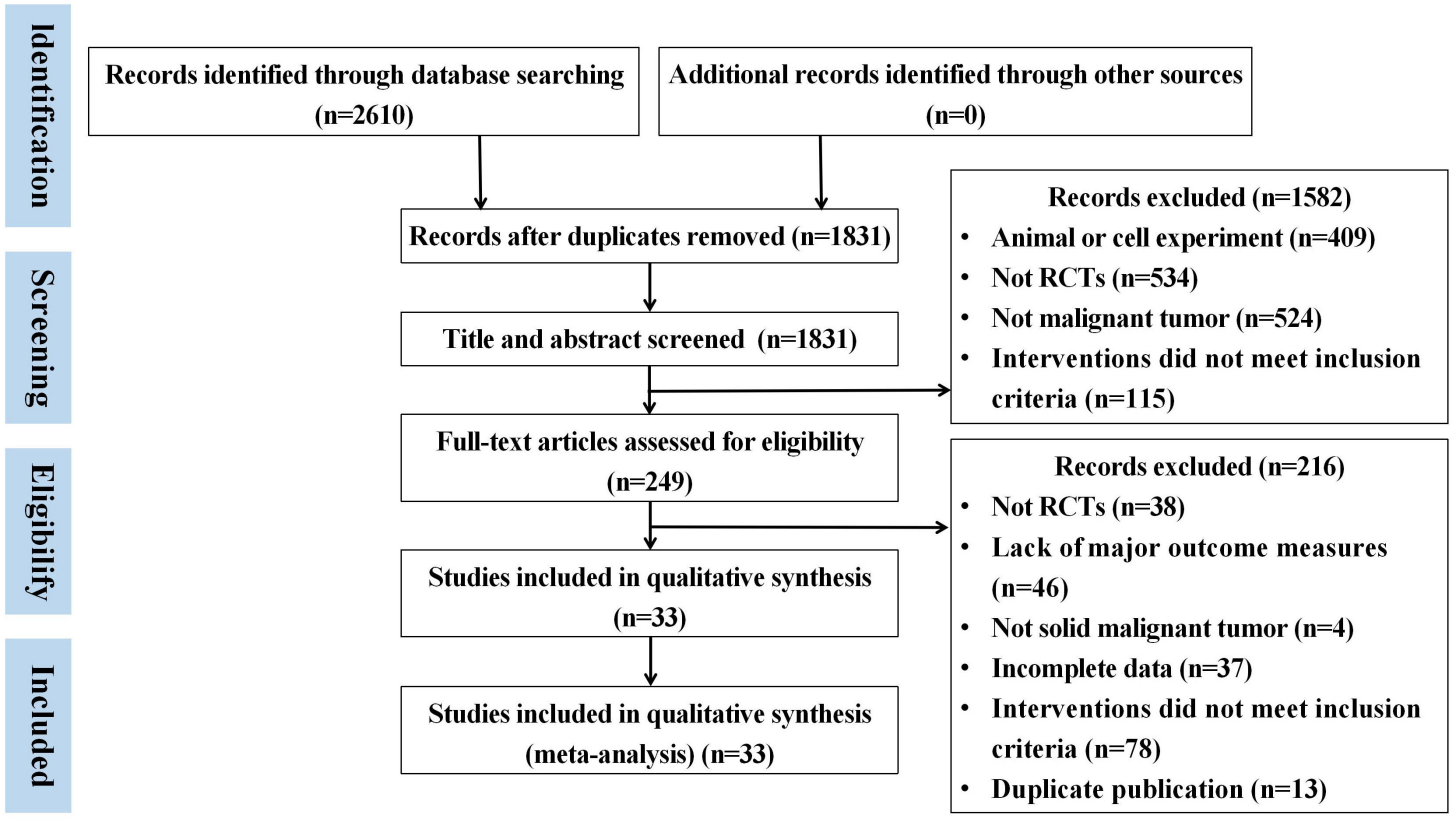
The specific retrieval processes summarized in a flow diagram.

A total of 2610 articles were obtained by searching the database. There were 779 duplicate literature were found. After reading the title and abstract, 1582 articles were excluded. Then, after strict literature screening and reading the full-text articles according to the inclusion and exclusion criteria, 216 articles were eliminated as follows: 38 did not RCTs, 78 did not meet the inclusion criteria, 46 studies lack of outcome measures, 37 had incomplete data, thirteen articles were published in duplicate and four were non-solid tumors. Finally, a total of 33 eligible trials were included. The specific retrieval process is shown in **Figure 1**.

### Study characteristics

Thirty-three RCTs were included with 2259 participants that divided into two groups that control group received conventional therapies (n=1130), and intervention groups received what control groups received plus acupuncture and/or moxibustion treatment (n=1129), including acupuncture, electroacupuncture, fire acupuncture and moxibustionon (19–51). Study characteristics of included studies was shown in **Table 1**. In participant, the average age of patients of the 33 studies were between 45 and 70 years old. There were 12 studies with lung cancer (20, 26, 29, 31, 33, 34, 37, 41, 46–49) and seven studies with gastrointestinal cancer (23, 30, 38, 39, 43–45). Nineteen studies used chemotherapy (19–28, 31, 33, 34, 37, 40–42, 46, 48), of which two studies combined with radiotherapy (24, 42) and two studies combined with TCM (20, 48). One study may have used one or more of the following: immunotherapy, targeted therapy, or chemotherapy (29). Eleven studies used acupuncture and moxibustion simultaneously (26, 28, 34, 36–38, 44, 45, 47, 48, 50). Fourteen studies used acupuncture only (19–24, 27, 31, 33, 39–42, 49), of which four studies used electroacupuncture (19, 22, 33, 39), two studies used fire acupuncture (31, 41). Moxibustion was used only in eight studies (25, 29, 30, 32, 35, 43, 46, 50). The most frequently used acupoints were: Zusanli (ST36), Qihai (CV6), Guanyuan (RN4), and Sanyinjiao (SP6). Study characteristics of included studies in **Table 1**.

**Table 1:** Study characteristics of included studies.

Study ID	Sample size (I/C)	Age (year) (I/C)		Cancer typology (I/C)		Clinical intervention (I/C)		Main point(s)	Duration of intervention	Outcome
Chen 2004 (19)	28/28	46.2	49	Nasopharyngeal cancer: n=11; NSCLC: n=17	Nasopharyngeal cancer: n=13; NSCLC: n=15	Electroacupuncture	Chemotherapy: CFP for nasopharyngeal cancer; VP for NSCLC	Zusanli (ST36)	42 days	②③④⑤
Chen 2008 (22)	30/30	62.07	60.8	NSCLC	NSCLC	Acupuncture	Chemotherapy and TCM treatment	Feishu (BL13); Zhongfu (LU13); Taiyuan (LU9); Fengmen (BL12); Xinshu (BL15); Gaohuangshu (BL43); Chize (LU5); Danzhong (RN17); Dazong; Tender point	42 days	②③
Chen 2016 (20)	30/30	50.9 ± 10.90	51.2 ± 7.63	Breast cancer	Breast cancer	Acupuncture	Chemotherapy and symptomatic supportive treatment	Neiguan (PC6); Shenmen (HT7); Guanyuan (RN4); Xuanzhong (GB39); Sanyinjiao (SP6); Zusanli (ST36); Xuehai (SP10);	24 days	②③⑤
Chen 2018 (21)	20/20	54.5 ± 4.6	55.7 ± 4.3	Breast cancer	Breast cancer	Electroacupuncture	Radical mastectomy for breast cancer and general anesthesia	Neiguan (PC6); Sanyinjiao (SP6); Hegu (LI4); Zusanli (ST36)	4 days	②③④⑤
Guo 2013 (23)	30/30	52.50 ± 13.21	49.17 ± 11.33	Gastric cancer	Gastric cancer	Acupuncture	Chemotherapy (DFP)	Zhongwan (CV12); Guanyuan (RN4); Neiguan (PC6); Zusanli (ST36)	42 days	②③④
Jiang 2020 (24)	44/45	49.62 ± 6.51	50.19 ± 6.82	Nasopharyngeal cancer	Nasopharyngeal cancer	Acupuncture	Radiotherapy 35 times and chemotherapy 3 times (DDP)	Tiantu (CV22); Renying (ST9); Guanyuan (RN4); Qihai (CV6)	49 days	②④⑥
Li 2009 (25)	30/30	64.10 ± 11.16	59.16 ± 12.87	Lung cancer: n=9; Intestinal cancer: n=7; Hepatocellular carcinoma: n=8; Gastric cancer: n=4; Breast cancer: n=2	Lung cancer: n=8; Intestinal cancer: n=8; Hepatocellular carcinoma: n=8; Gastric cancer: n=3; Breast cancer: n=3	Moxibustionon	Chemotherapy	Gaohuangshu (BL43); Pishu (BL20); Weishu (BL21); Geshu (BL17); Shenshu (BL23)	14 days	②③⑤
Li 2020 (26)	32/32	65.12 ± 5.18	65.85 ± 5.02	NSCLC	NSCLC	Acupuncture and moxibustionon	Chemotherapy (AP)	Zusanli (ST36); Qihai (CV6)	84 days	②③④
Liao 2018 (27)	29/30	48.72 ± 1.23	48.80 ± 1.31	Lung cancer: n=20; Intestinal cancer: n=3; Gastric cancer: n=3; Breast cancer: n=2; Cervical cancer: n=1	Lung cancer: n=17; Intestinal cancer: n=2; Gastric cancer: n=2; Endometrial cancer: n=2; Nasopharyngeal cancer: n=2; Ovarian cancer:n=3; Cervical cancer: n=1; Thymoma: n=1	Acupuncture	Chemotherapy (Platinum-containing)	Zusanli (ST36); Zhigou (TE6); Taichong (LR3); Taibai (SP3); Xinmen; Xiaotianxin	10 days	②③④
Liu 2022 (28)	30/30	55.10 ± 9.52	55.57 ± 9.12	Lung cancer: n=11; Gastric cancer: n=7; Ovarian cancer: n=4; Colorectal cancer: n=6; Other: n=2	Lung cancer: n=10; Gastric cancer: n=5; Ovarian cancer: n=5; Colorectal cancer: n=7; Other tumors: n=3	Acupuncture and moxibustionon	Chemotherapy and symptomatic treatment	Shenshu (BL23); Pishu (BL20); Ganshu (BL18)	10 days	①②③④
	30/30	57.57 ± 8.90	58.23 ± 8.16	Lung cancer: n=10; Gastric cancer: n=4; Ovarian cancer: n=3; Colorectal cancer: n=9; Other tumors: n=4	Lung cancer: n=12; Gastric cancer: n=2; Ovarian cancer: n=2; Colorectal cancer: n=10; Other tumors: n=4	Acupuncture and moxibustionon	Chemotherapy and interleukin-11	Shenshu (BL23); Pishu (BL20); Ganshu (BL18)	10 days	
Mao 2022 (29)	17/16	65.41 ± 11.56	67.94 ± 10.08	NSCLC	NSCLC	Moxibustionon	Pembrolizumab, pemetrexed, Bevacizumab (1 or more)	Zusanli (ST36)	21 days	②⑤
Pan 2013 (30)	31/30	57	58	Gastric cancer	Gastric cancer	Moxibustionon	TCM treatment	Zusanli (ST36)	6 months	②③⑤
Pei 2016 (31)	30/30	58.90 ± 8.73	63.00 ± 8.51	NSCLC	NSCLC	Fire acupuncture	Chemotherapy (TP/GP/DP/NP)	Geshu (BL17); Danshu (BL19)	21 days	②③⑤⑥
Qin 2012 (32)	15/15	N	N	Gastric cancer: n=3; Intestinal cancer: n=6; Lung cancer: n=1; Ovarian cancer: n=2; Liver cancer: n=3	Gastric cancer: n=4; Intestinal cancer: n=7; Lung cancer: n=2; Ovarian cancer: n=1; Liver cancer: n=2	Moxibustionon	Symptomatic treatment (including TCM treatment)	Shenque (RN8); Guanyuan (RN4); Zhongwan (CV12); Qihai (CV6); Tianshu (ST25); Zusanli (ST36); Yongquan (KI1)	14 days	②③④⑤
Wang 2016 (33)	70/70	60.57 ± 5.33	60.84 ± 5.41	NSCLC	NSCLC	Electroacupuncture	Chemotherapy (GP)	Zusanli (ST36); Sanyinjiao (SP6)	21 days	②③⑤
Wang 2023 (34)	30/30	62.43 ± 7.62	60.81 ± 6.77	NSCLC	NSCLC	Acupuncture and moxibustionon	Chemotherapy (TP/GP/AP)	Qihai (CV6); Guanyuan (RN4); Zusanli (ST36); Pishu (BL20); Shenshu (BL23)	21 days	②③④
Wu 2016 (35)	20/20	62.04 ± 7.783	64.40 ± 8.829	Esophageal cancer	Esophageal cancer	Moxibustionon	N	Feishu (BL13); Gaohuangshu (BL43); Zusanli (ST36)	42–49 days	①②③④
Wu 2021 (36)	41/41	48.72 ± 4.16	49.01 ± 4.14	Breast cancer: n=9; Lung cancer: n=14; Gastric cancer: n=12; Liver cancer: n=3; Other tumors: n=3;	Breast cancer: n=8; Lung cancer: n=15; Gastric cancer: n=10; Liver cancer: n=5; Other tumors: n=3	Acupuncture and moxibustionon	Symptomatic treatment	Zusanli (ST36); Sanyinjiao (SP6)	14 days	②④
Wu 2022 (37)	50/50	50.78 ± 7.32	50.85 ± 7.24	NSCLC	NSCLC	Acupuncture and moxibustionon	Chemotherapy (TP)	Xuehai (SP10); Waiguan (SI4); Taichong (LR3)	14 days	①②
Xiang 2022 (38)	34/34	57.30 ± 8.26	57.25 ± 8.22	Gastric cancer: n=12; Rectal cancer: n=11; Colon cancer: n=11	Gastric cancer: n=13; Rectal cancer: n=10; Colon cancer: n=11	Acupuncture and moxibustionon	TCM treatment	Zusanli (ST36); Qihai (CV6)	14 days	②③④
Xing 2022 (39)	29/29	68.48 ± 5,37	69.45 ± 5,89	Gastric cancer	Gastric cancer	Electroacupuncture	Surgery with transversus abdo minis plane block anesthesia	Hegu (LI4); Neiguan (PC6); Zusanli (ST36)	1 days	②③④
Xiong 2017 (40)	38/38	65.43 ± 7.86	65.57 ± 7.91	Liver cancer	Liver cancer	Acupuncture	Hepatic Chemoembolization (floxuridine, epirubicin and hydroxycamptothecin)	Baihui (GV20); Neiguan (PC6); Sanyinjiao (SP6); Weiqu (in head skin point)	5 days	②③④⑤
Xu 2012 (41)	30/30	61.57 ± 6.53	61.63 ± 6.55	NSCLC	NSCLC	Fire acupuncture	Chemotherapy (TP/GP)	Geshu (BL17); Danshu (BL19)	7 days	②③④
Xu 2024 (42)	30/30	51.60 ± 9.45	50.73 ± 9.18	Cervical cancer	Cervical cancer	Acupuncture	Radiotherapy and chemotherapy 5 times (DDP)	Guanyuan (RN4); Qihai (CV6)	35 days	②③④⑥
Xue 2013 (43)	32/32	57.4 ± 6.6		Gastric cancer	Gastric cancer	Moxibustionon	Conventional nursing and TCM treatment	Zusanli (ST36)	6 months	②③⑤
Yang 2021 (44)	33/32	54.38 ± 8.97	54.38 ± 8.97	Colorectal cancer	Colorectal cancer	Acupuncture and moxibustionon	Symptomatic supportive treatment	Guanyuan (RN4); Qihai (CV6); Tianshu (ST25); Taixi (KI3); Zusanli (ST36)	28 days	②③④⑤
Zhang 2011 (45)	35/35	57.1 ± 11.7	59.1 ± 8.5	Colorectal cancer	Colorectal cancer	Acupuncture and moxibustionon	Postoperative routine treatment	Zusanli (ST36); Shangjuxu (ST37); Xiajuxu (ST39); Sanyinjiao (SP6); Yinlingquan (SP9)	10 days	②④⑤
Zhang 2017 (46)	30/30	57.27 ± 5.38	57.32 ± 5.36	NSCLC	NSCLC	Moxibustionon	Chemotherapy (GP)	Zusanli (ST36); Guanyuan (RN4); Qihai (CV6); Feishu (BL13); Shenshu (BL23); Pishu (BL20); Gaohuangshu (BL43)	21 days	②③④⑥
Zhang 2021 (47)	66/66	50.77 ± 3.19	51.02 ± 2.48	NSCLC	NSCLC	Acupuncture and moxibustionon	Conventional nursing	Sanyinjiao (SP6); Guanyuan (RN4); Zusanli (ST36); Hegu (LI4)	30 days	①②
Zhao 2022 (48)	34/34	47.13 ± 4.06	45.47 ± 4.70	NSCLC	NSCLC	Acupuncture and moxibustionon	Chemotherapy (Taxol and Platinum)and TCM treatment	Zusanli (ST36); Zhongwan (CV12); Danzhong (RN17); Qihai (CV6)	21 days	②③⑤⑥
Zhao 2023 (49)	32/33	64.25 ± 7.81	64.27 ± 9.65	Lung Cancer	Lung Cancer	Acupuncture	Symptomatic treatment	Danzhong (RN17); Zhongwan (CV12); Qihai (CV6); Zusanli (ST36); Xuehai (SP10); Waiguan (SI4)	N	②③④
Zhou 2019 (50)	30/30	67 ± 8	63 ± 11	Lung cancer: n=7; Digestive system tumors: n=15; Gynecological tumors: n=5; Other tumors: n=2	Lung cancer: n=12; Digestive system tumors: n=11; Gynecological tumors: n=2	Moxibustionon	Comprehensive treatment of traditional Chinese and western medicine	Zusanli (ST36)	12 days	②③④
Zhu 2023 (51)	40/40	46.18 ± 10.13	45.70 ± 10.07	gastric cancer: n=16; Cervical cancer: n=10; Rectal cancer: n=8; Colon cancer: n=6	gastric cancer: n=18; Cervical cancer: n=8; Rectal cancer: n=7; Colon cancer: n=7	Acupuncture and moxibustionon	Gastrointestinal decompression	Zhongwan (CV12); Xiawan; Shangjuxu (ST37); Xiajuxu (ST39); Tianshu (ST25); Sanyinjiao (SP6); Zusanli (ST36)	Until 1 week after the patient’s intestinal obstruction improved	②⑤

I, Intervention group; C, Control group; NSCLC, Non-small Cell Lung Cancer; DDP, Cisplatin; C, Cyclophosphamide; F, 5 fluorouracil; V, Etoposide; TP, Taxol and Cisplatin; GP, Gemcitabine and Cisplatin; DP, Docetaxel and Cisplatin; NP, Vinorelbine and Cisplatin; AP, Pemetrexed and Cisplatin; TCM, Traditional Chinese Medicine; N, None.

①CD3+; ②CD4+; ③CD8+; ④CD4+/CD8+; ⑤NK; ⑥clinical efficacy.

### Methodological quality of included studies

**Figure 2:**
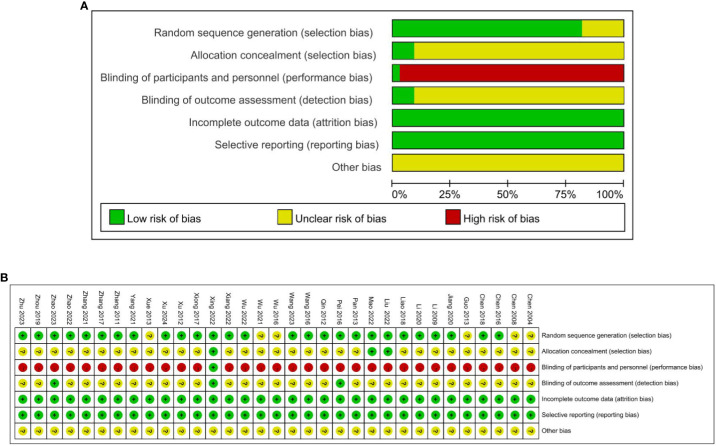
**(A)** Quality assessment of the included trials risk of bias graph. **(B)** Quality assessment of the included trials risk of bias graph.

Twenty-seven RCTs were assessed as low risk for random sequence (21, 22, 24–34, 37–42, 44–51), including 24 RCTs used the random number table method (22, 24–32, 34, 37–40, 42, 44–51), one RCT used the simple randomization (41), two RCTs used the block randomization (21, 33) and the other six RCTs did not elaborate on specific methods of randomization (19, 20, 23, 35, 36, 43), so the risks were unclear. Due to the particularness of acupuncture and moxibustion, it is difficult to blind the practitioners of acupuncture and moxibustion. One study mentioned the blinding of researchers and patients (39), one study mentioned the blinding of statisticians and examiners (31), and one study mentioned the separation of researchers, data collection, and data statistical analysis (49), which considered low risk of bias. Three of the included studies achieved concealment by using sealed envelopes and were deemed to be at low risk of bias, which resulted in a low risk of bias in relative fields (28, 29, 39). None of the 33 studies had missing data or missing data that were comparable in each intervention group, and the reasons for missing data were similar, so they were rated as having a low attrition risk of bias. All 33 studies had a low risk of reporting bias. The quality assessment of the included trials risk of bias graph in **Figure 2**.

### Meta-analysis results

#### CD3^+^

All the included RCTs reported the CD3+ T lymphocyte counts of the patients. Meta-analysis results showed that: effect of the intervention group was significantly better than that of the control group in improving the CD3+ (MD = 4.97, 95% CI 3.81 to 6.13). Further subgroup analysis was conducted according to cancer typology, two groups clinical intervention (including combined chemotherapy, surgery, and other therapies), acupoint selection (according to whether the use of Zusanli (ST36) acupoint is divided), intervention group clinical treatment (according to the use of acupuncture, moxibustion, acupuncture and moxibustion divided) and duration of treatment (according to treatment duration of 30 days or more and less than 30 days divided). Subgroup analysis showed no statistical difference in cancer typology (P=1.00), control group clinical intervention (P=0.16), acupoint selection (P=0.64) and duration of treatment (P=0.53). The forest plot was shown in **Supplementary Materials**. (**Supplementary Figures S1**–**S4**).

**Figure 3:**
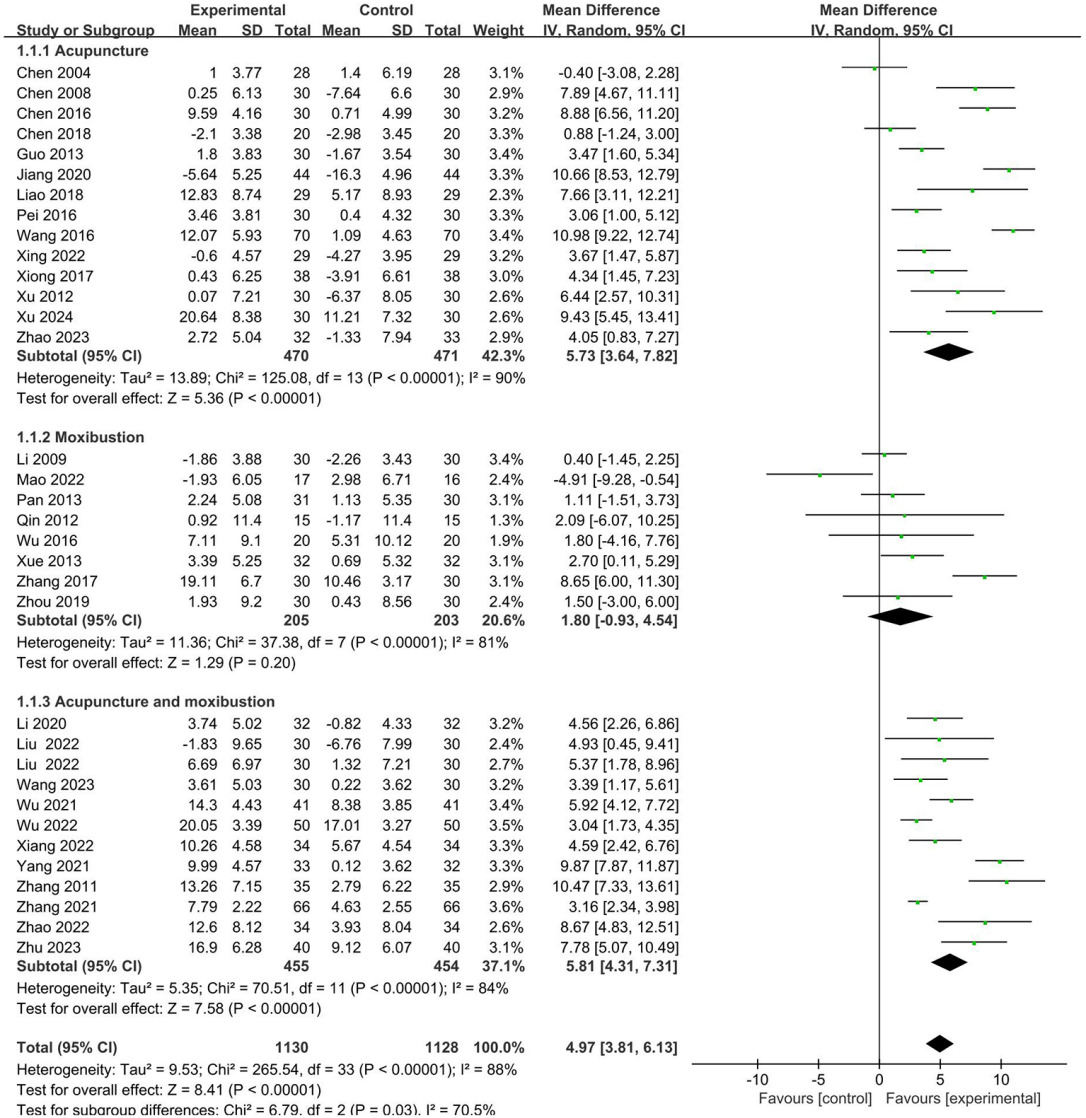
Forest plot for the CD3^+^ level of malignant tumors patients (n=33).

The subgroup analysis revealed that the intervention group clinical treatment (P = 0.03) were the main source of heterogeneity in CD3^+^ and the forest plot was shown in **Figure 3**. No source of heterogeneity was identified by sensitivity analysis.

#### CD4^+^

A total of 33 studies (19–51) analyzed the effects of CD4^+^. The results showed that the intervention group had a more significant effect on increasing the CD4^+^ (MD = 4.25, 95% CI 2.80 to 5.69). Further subgroup analysis was conducted. It is shown that there is no statistical difference in cancer typology (P=0.23), two groups clinical intervention (P=0.80), acupoint selection (P=0.40) and duration of treatment (P=0.39). The forest plot was shown in **Supplementary Materials**. (**Supplementary Figures S5**–**S8**).

**Figure 4:**
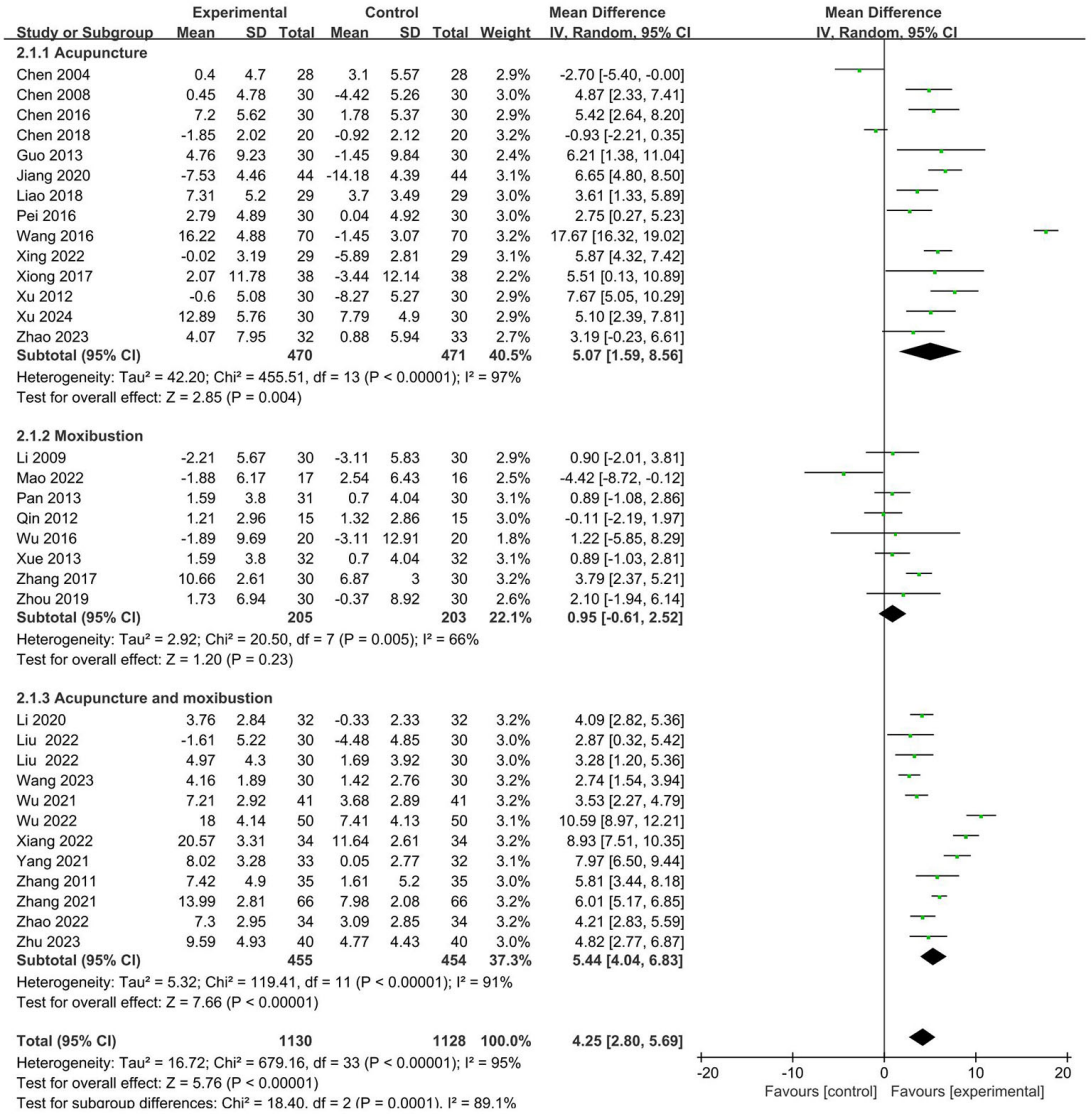
Forest plot for the CD4^+^ of level of malignant tumors patients (n=33).

Similarly, the subgroup analysis revealed that the intervention group clinical treatment (P = 0.0001) were the main source of heterogeneity in CD4^+^. Forest plot was shown in **Figure 4**. No source of heterogeneity was identified by sensitivity analysis.

#### CD8^+^

**Figure 5:**
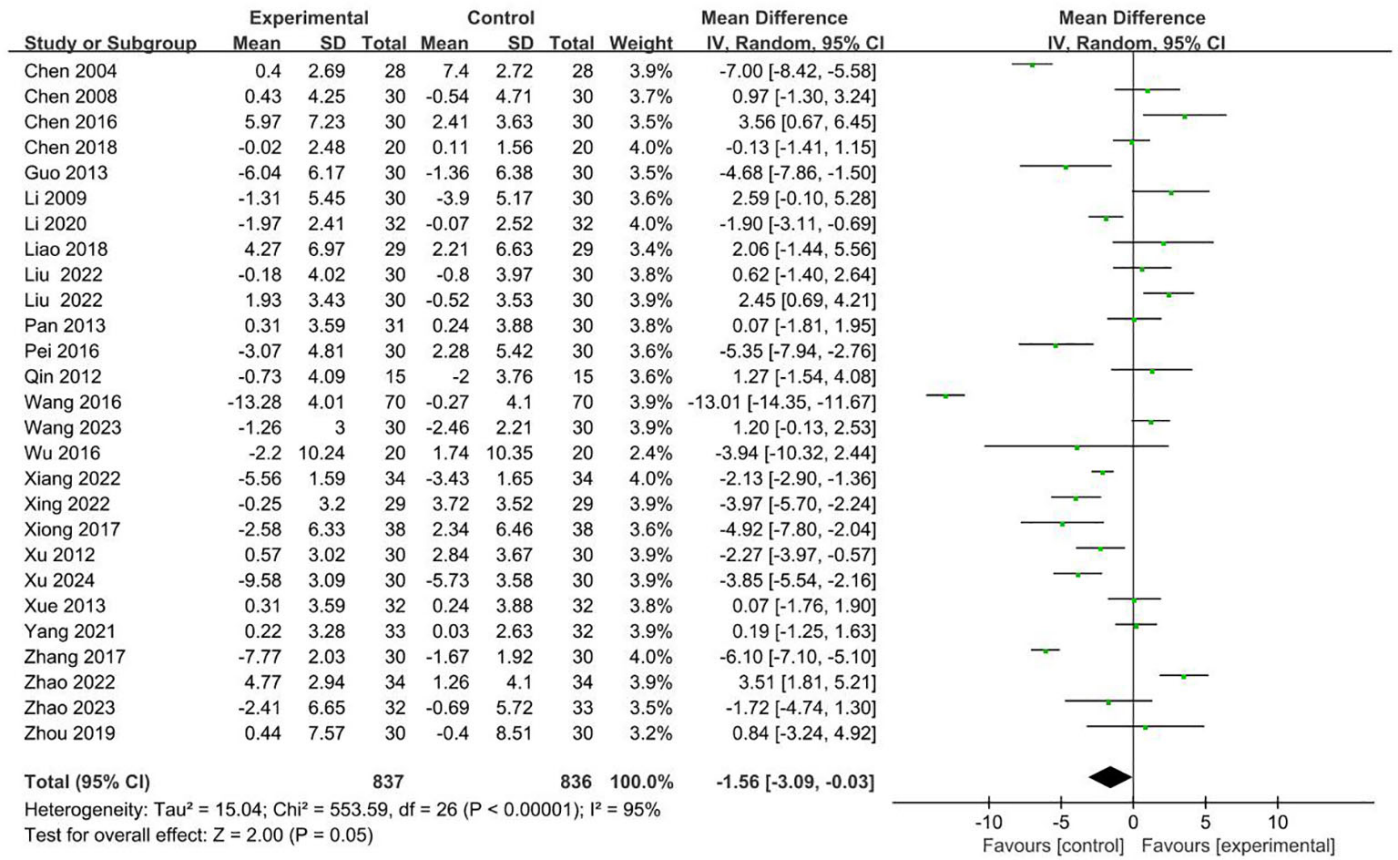
Forest plot for the CD8^+^ level of malignant tumors patients (n=26).

26 RCTs with 1673 patients reported CD8^+^ index (19–23, 25–28, 30–35, 38–43, 45, 46, 48–50). Meta-analysis showed that the intervention group was decreased compared with the control group in CD8^+^ (MD = -1.56 95% CI -3.09– -0.03). The results are presented in **Figure 5**.

No sources of heterogeneity were identified by subgroup analysis in the intervention group clinical treatment (P = 0.07), cancer typology (P=0.37), two groups clinical intervention (P=0.48), acupoint selection (P=0.72) and duration of treatment (P=0.45). (**Supplementary Figures S9**–**S13**).

#### CD4^+^/CD8^+^

**Figure 6:**
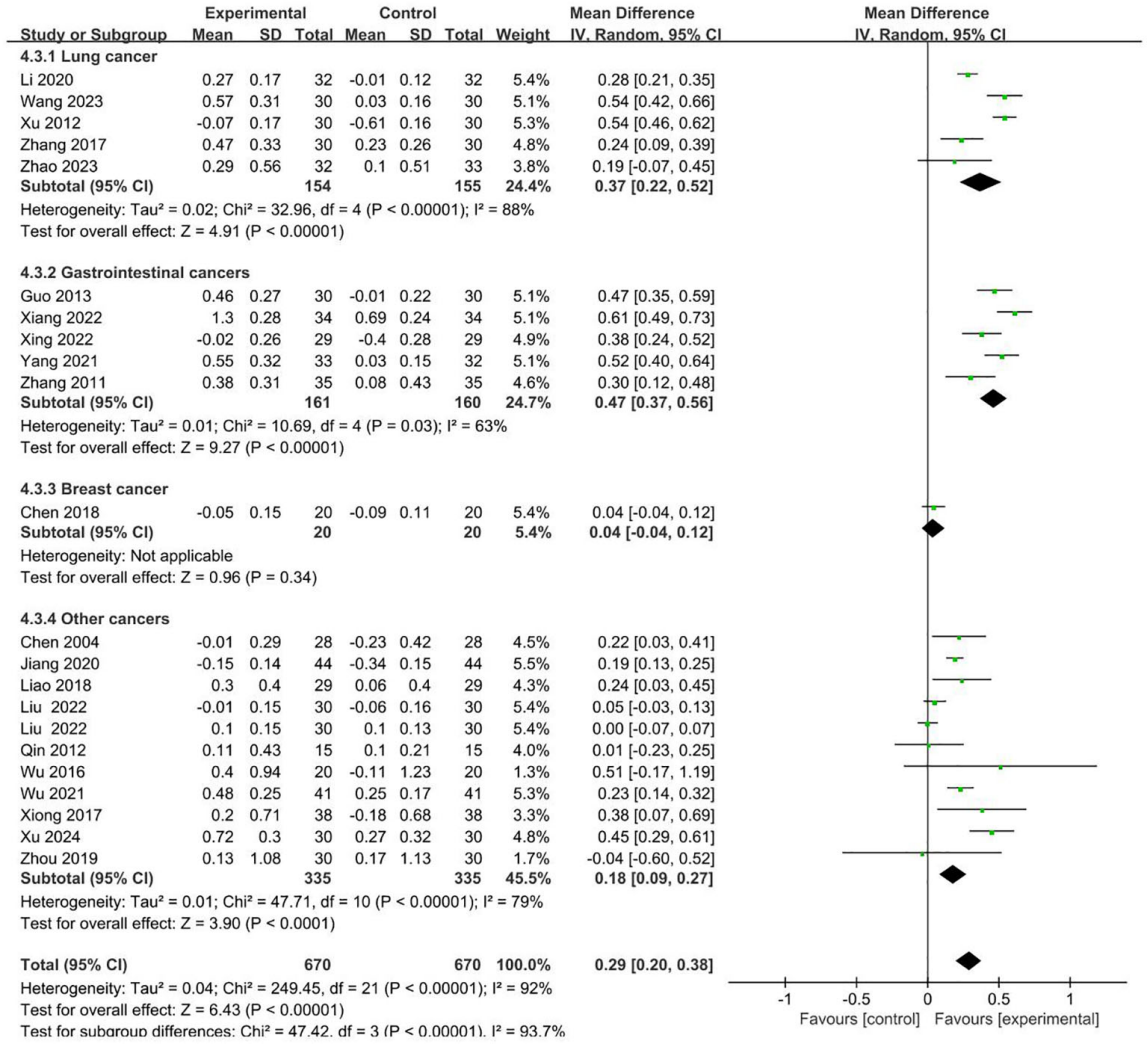
Forest plot for the CD4^+^/CD8^+^ level of malignant tumors patients (n=21).

21 RCTs involving 670 cases in the intervention group and 670 cases in the control group reported CD4^+^/CD8^+^ in the outcome indicators (19, 22–24, 26–28, 32, 34–36, 38–42, 44–46, 49, 50). Meta-analysis showed that compared to control groups, intervention groups were significantly better in increasing the level of CD4^+^/CD8^+^ (MD = 0.29 95% CI 0.20–0.38). Subgroup analysis showed that the heterogeneity was associated with the cancer typology (P<0.00001). The results are presented in **Figure 6**.

No sources of heterogeneity were identified by subgroup analysis in the intervention group clinical treatment (P = 0.31), two groups clinical intervention (P=0.85), acupoint selection (P=0.63) and duration of treatment (P=0.65). (**Supplementary Figures S14**–**S17**).

#### NK

**Figure 7:**
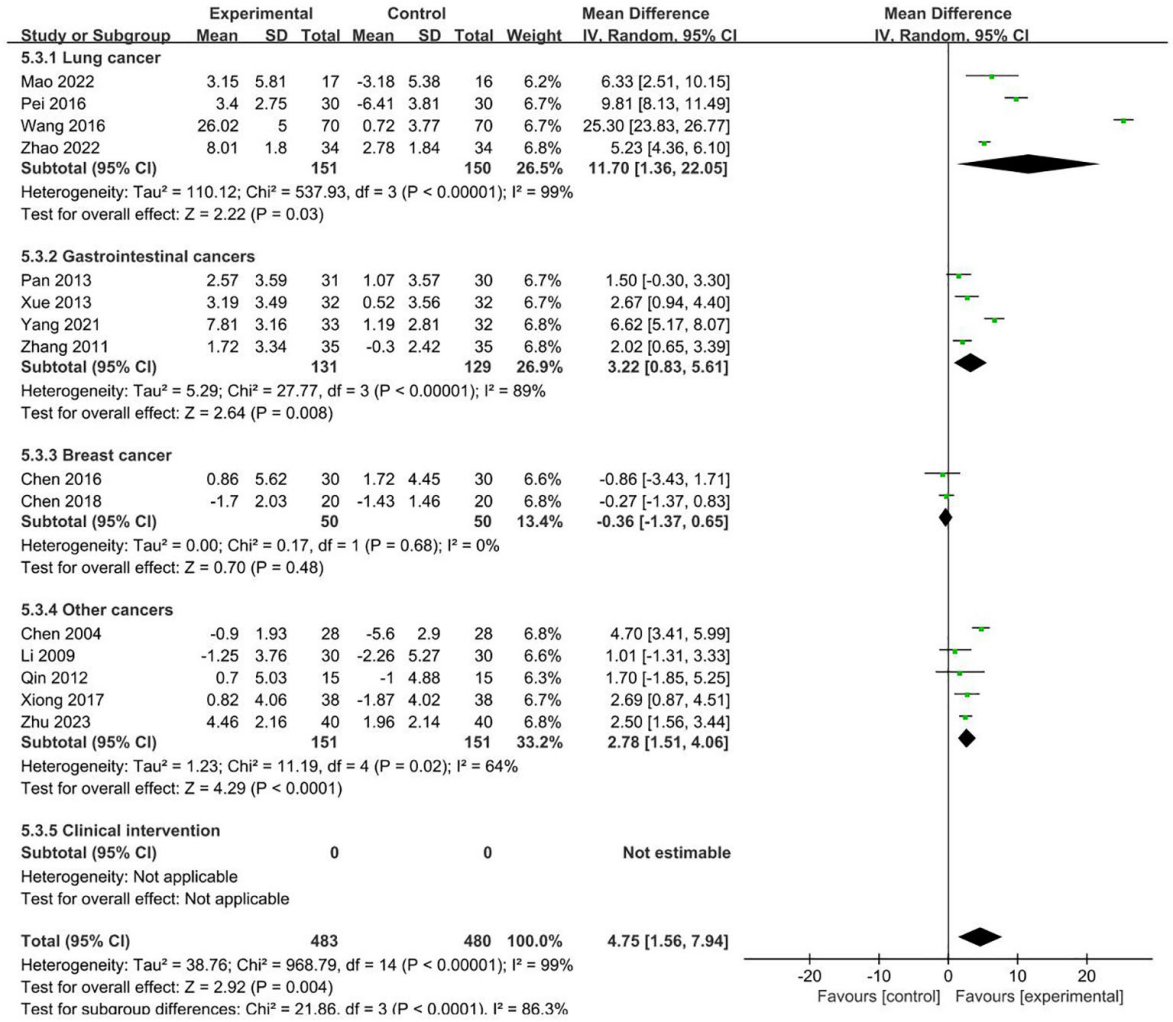
Forest plot for the NK level of malignant tumors patients (n=15).

**Figure 8:**
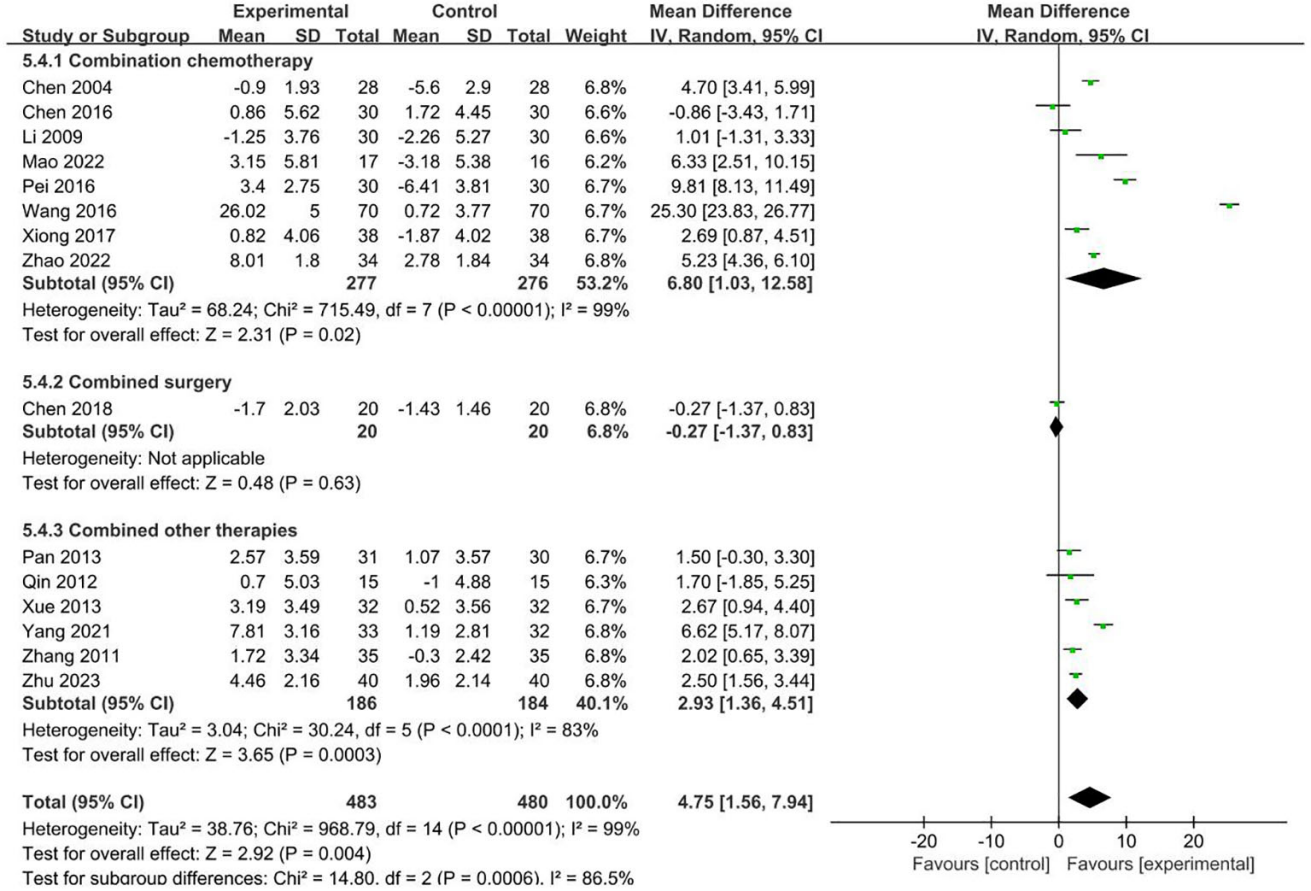
Forest plot for the NK level of malignant tumors patients (n=15).

15 RCTs with 963 patients reported NK index (19, 21, 22, 25, 29–33, 40, 43–45, 48, 51). Meta-analysis showed that the intervention group was greatly improved compared with the control group in increasing NK (MD = 4.75, 95% CI 1.56–7.94). Subgroup analysis showed that the heterogeneity was associated with the cancer typology (P<0.0001) and two groups clinical intervention (P=0.0006). The results are presented in **Figures 7**, **8**.

No sources of heterogeneity were identified by subgroup analysis in the intervention group clinical treatment (P = 0.21), acupoint selection (P=0.94) and duration of treatment (P=0.34). (**Supplementary Figure S18**–**S20**).

#### Clinical effective rate

**Figure 9:**
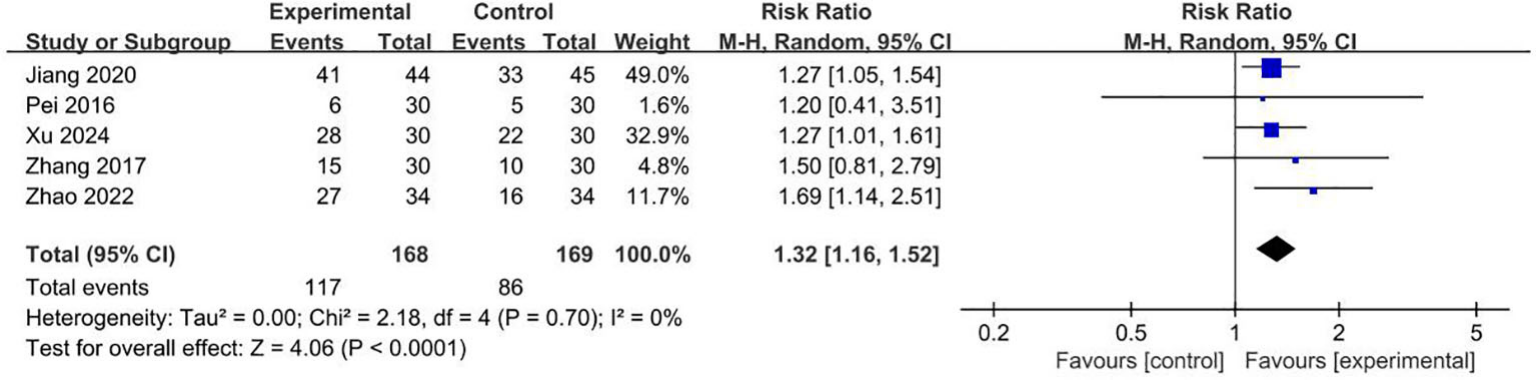
Forest plot for the clinical effective rate of malignant tumors patients (n=5).

Five RCTs with 337 patients reported clinical efficacy in outcome indicators (24, 31, 42, 46, 48). The results showed that compared to the patients who received conventional therapies, those who received acupuncture and moxibustion plus conventional therapies have a significantly better clinical efficacy (RR = 1.32, 95% CI 1.16–1.52). The forest plot is shown in **Figure 9**.

### Publication bias

**Figure 10:**
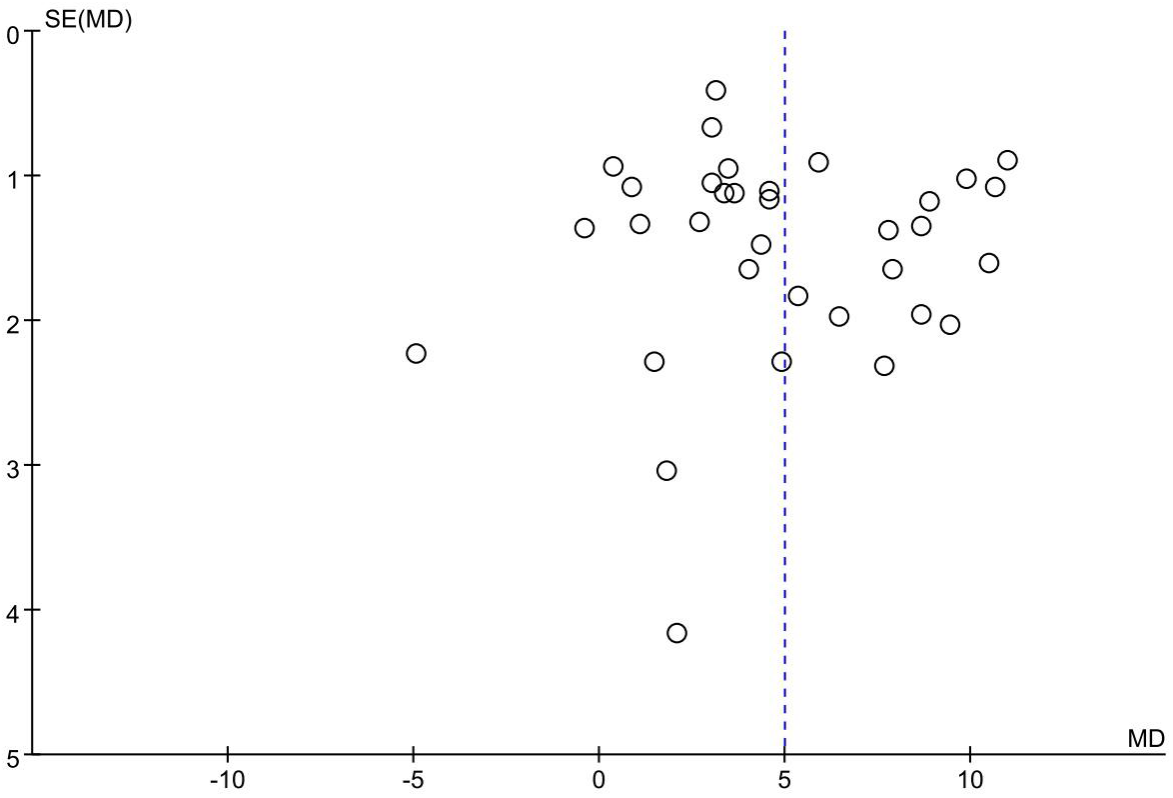
Funnel plot showing publication bias; CD3^+^.

**Figure 11:**
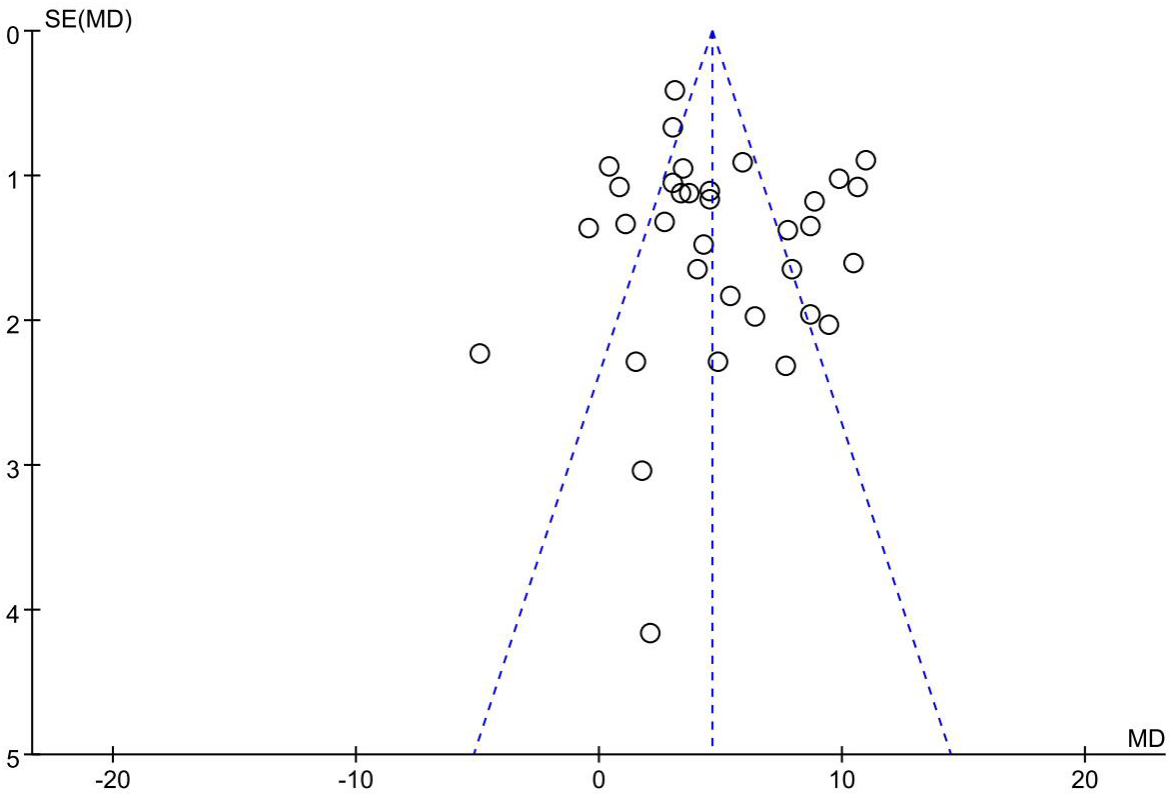
Funnel plot showing publication bias; CD4^+^.

The funnel plot of the primary outcome (CD3^+^, CD4^+^) displayed an uneven distribution of studies, suggesting presence of publication bias. The result is presented in **Figures 10**, **11**. The publication bias may be associated with negative results not being published and a part of studies had small sample sizes.

## Discussion

The results showed that combined acupuncture and moxibustion, the levels of CD3^+^, CD4^+^, CD4^+^/CD8^+^, and NK cells increased, while the level of CD8^+^ cells decreased. Additionally, acupuncture and moxibustion indicated a positive effect on short-term clinical outcomes.

T lymphocytes cells are an important component of the immune system and are the main cellular component of the adaptive immune system, playing a crucial role in resisting pathogen invasion and suppressing tumorigenesis (52). A large number of studies have shown that T lymphocytes in the tumor microenvironment play an important role in the anti-tumor response. High levels of tumor-infiltrating T cells usually indicate a better prognosis for patients (53–55). CD3+ can effectively reflect the levels of CD4^+^ and CD8^+^. CD4^+^ directly reflects the immune function of the patient (56). CD8^+^ participates in the process of transmitting activation signals from T lymphocyte receptors recognizing antigens, and it belongs to cytotoxic T lymphocytes. CD8^+^ can produce negative regulation on T lymphocyte and B lymphocyte function through its own and related cytokines, inhibiting the formation of cellular immunity and antibodies, and its increased level can aggravate the immune dysregulation (57). CD4^+^/CD8^+^ balance is a key factor in maintaining the immune response (58). NK cells are an important component of tumor immune surveillance and play an important role in preventing tumor growth (59).

However, it has been proven that CD8^+^ T lymphocytes are associated with poor prognosis in cancer patients. As the tumor grows and develops, it can stimulate and induce the proliferation of CD8^+^ T lymphocytes, and the increase of CD8^+^ T lymphocytes can promote the growth of tumor cells to a certain extent, increase the risk of metastasis and recurrence, and is not conducive to the prognosis (60), and most patients exhibit a decrease in CD3^+^ and CD4^+^, leading to an imbalance of CD4^+^/CD8^+^ and a predominance of cell-mediated immune negative effects (61). The study by Muhammad Ramzan et al. showed that high infiltration of CD8^+^ cells in tumor tissue suggests a high recurrence rate and poor prognosis in HCC patients (62). Similarly, in colorectal cancer patients, studies have shown that high CD8^+^ T lymphocyte content may be associated with adverse clinical outcomes, and a decrease in CD4^+^ T lymphocyte content and a decrease in the CD4^+^/CD8^+^ ratio indicate that CRC is in a progressive state and undergoing accelerated proliferation (63). In lung cancer and melanoma patients, CD8^+^ T cell levels are low before treatment and ultimately derive a durable benefit from immunotherapy (64, 65).

The possible reasons for CD8^+^ cell elevation being associated with poor prognosis in patients are as follows: in the context of long-term suppressive tumor microenvironment, tumor-specific CD8^+^ T lymphocytes are prone to enter the “T lymphocyte exhaustion of function” stage (66–68), characterized by increased expression of immune inhibitory receptors such as lymphocyte activation gene 3 (LAG-3), cytotoxic T lymphocyte-associated antigen 4 (CTLA-4), and T cell immunoglobulin 3 (TIM-3) on the surface of lymphocytes, impaired production of cytokines such as IL-2, TNFα, and IFNγ, and impaired tumor killing ability (69, 70). Relevant studies show that the increase of CD8 ^+^T cells may be related to the mechanism of tumor immune escape (71). This also suggests that in the future further researches on the effect of acupuncture on CD8^+^ cell function are needed.CD4^+^ cells and CD8^+^ cells are mutually inducing and restraining, forming a network of cells that is important for regulating immune responses and maintaining immune homeosta (72). When the CD8^+^ cells increases, the ratio of the CD4^+^ and CD8^+^ cells changes, it can cause the immune function of the body to decrease, thereby weakening the anti-tumor ability of the body (73). Meanwhile, CD8^+^ T cells have cytotoxic effects on antigen presenting cells, they can also inhibit the anti-tumor effect of CD4^+^ cells by producing inhibitory cytokines (74).

Furthermore, a compelling body of clinical evidence reveals that these traditional therapies can significantly improve the humoral immune levels and cytokine profiles of cancer patients. For instance, research has demonstrated that acupuncture and moxibustion can elevate key humoral immune markers such as IgM, IgG, C3, and C4 (48) and reduce TNF-α, TGF-β1 levels, increase IL-2 levels in tumor patients (24, 33, 41, 42), suggesting a potential mechanism for bolstering the body’s immune defenses against cancer.

Subgroup analysis showed that different acupuncture and moxibustion methods were one of the main sources leading to heterogeneity of CD3^+^ and CD4^+^. The heterogeneity of CD4^+^/CD8^+^ and NK cells is caused by different types of cancer, and the heterogeneity of NK cells is also caused by different stages of treatment.

In the field of clinical research, the selected acupoints and the determined treatment duration exhibit a relatively diverse range of characteristics. This study focuses on conducting subgroup analyses based on two key factors: whether the Zusanli acupoint is selected and whether the treatment duration exceeds 30 days. The analysis results indicate that these two factors are not the root causes of the heterogeneity in this study. However, the clinical issue of how to select the optimal acupoints and determine the optimal treatment duration to effectively enhance the immune function of cancer patients remains at a stage that requires in-depth research and exploration. More high-quality research results are needed to provide strong evidence and references.

### Limitations

The meta-analysis has several limitations. Due to limited resources, this study only retrieved eight databases of published studies, which were all in Chinese or English. All included patients were from China. The single - source samples inevitably caused racial and genetic bias. They can’t adequately represent the diversity and complexity in disease features and genetic background across different races. This may limit the generalizability of the research findings. In addition, the majority of the included studies had small sample sizes, which may limit the persuasiveness of the results to some extent. Due to the uniqueness of acupuncture and moxibustion, all trials included in the study were not blinded to the acupuncturis. None of the 33 studies followed patients for a long time, so we cannot know the long-term effects of acupuncture and moxibustion on cancer patients. In the publication bias section, there was publication bias in CD3^+^ and CD4^+^ studies. This bias may be related to negative results not being published.

### Implications for future research and clinical practice

In clinical practice, acupuncture and moxibustion can be used as an auxiliary therapeutic measure for patients who can accept it. Based on the above limitations, more large-sample, multi-center and more diverse participant recruitment clinical trials are needed in the future. By including individuals from various racial and ethnic backgrounds, researchers can obtain a broader and more representative dataset. In the study design, strict prospective design methods should be used to ensure the quality of outcomes, especially the blind setting and long-term follow-up of outcome indicators. Only one of the studies included partial patients who combined immunotherapy, and the clinical efficacy of acupuncture therapy to combine immunotherapy needs to be further confirmed by more research. In addition, the optimal acupuncture intervention duration and frequency for enhancing immune function need to be further explored in future studies.

## Conclusion

In summary, in this systematic review and meta-analysis of 33 trials, including 1,378 patients with malignant tumors, acupuncture and moxibustion was found to have statistically significant and clinically meaningful effects on improving immune function compared to no acupuncture and moxibustion.

## Data Availability

The datasets presented in this study can be found in online repositories. The names of the repository/repositories and accession number(s) can be found in the article/**Supplementary Material**.
