# Correction: Effect of acupuncture and moxibustion on the immune function of patients with malignant tumors: a systematic review and meta-analysis

**DOI:** 10.3389/fimmu.2025.1685492

**Published:** 2025-10-10

**Authors:** Yan Wang, Bailu Sui, Ying Zhang, Liyuan Fang, Yi Xie, Yuhang Fang, Runxi Wang

**Affiliations:** ^1^ Department of Oncology, Guang’anmen Hospital, China Academy of Chinese Medical Sciences, Beijing, China; ^2^ Graduate School, Beijing University of Chinese Medicine, Beijing, China

**Keywords:** acupuncture, moxibustion, T lymphocyte cells, malignant tumors, meta-analysis

The following statement was erroneously omitted for the authors Wang Yan and Sui Bailu: “†These authors have contributed equally to this work and share first authorship.”

In the published article, an incorrect **Funding** statement was provided. The corrected **Funding** statement appears below.

“This work was supported by High Level Chinese Medical Hospital Promotion Project - Traditional Chinese Medicine Clinical Evidence-based Research Special Project - Clinical Efficacy Evaluation and Mechanism Research of Potential Advantage Diseases of Acupuncture (HLCMHPP2023089).”

The original version of this article has been updated.

