# Supplementary material for: Effect of acupuncture and moxibustion on the immune function of patients with malignant tumors: a systematic review and meta-analysis
Source: Front Immunol. 2025 Jul 25;16:1583522. doi: 10.3389/fimmu.2025.1583522 (PMC12331736; doi:10.3389/fimmu.2025.1583522)
Supplement: Supplementary file 1 [file DataSheet1.docx]

**Supplementary File 1.**

**Forest plots for subgroup analysis**


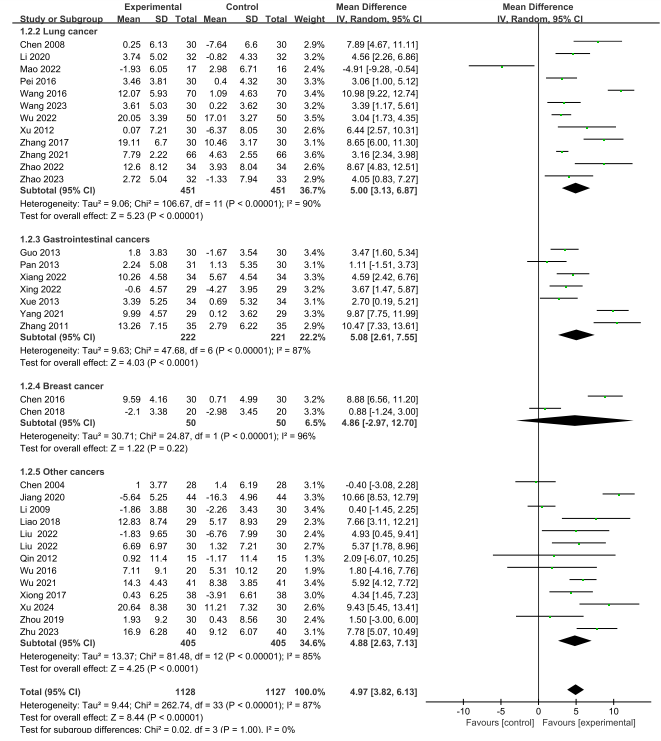


Figure S1 Forest plot for the CD3^+^ level of malignant tumors patients (n=33)

Subgroup analysis was conducted according to cancer typology of CD3^+^


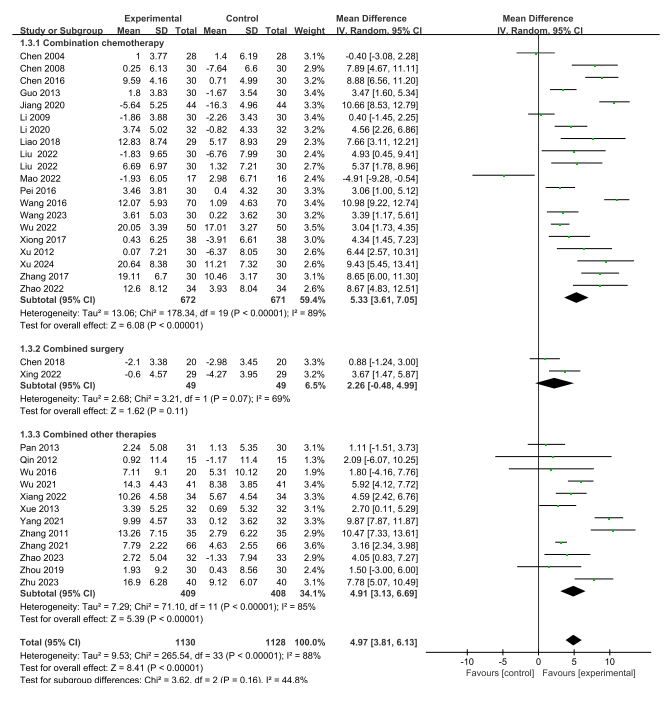


Figure S2 Forest plot for the CD3^+^ level of malignant tumors patients (n=33)

Subgroup analysis was conducted according to clinical intervention of CD3^+^


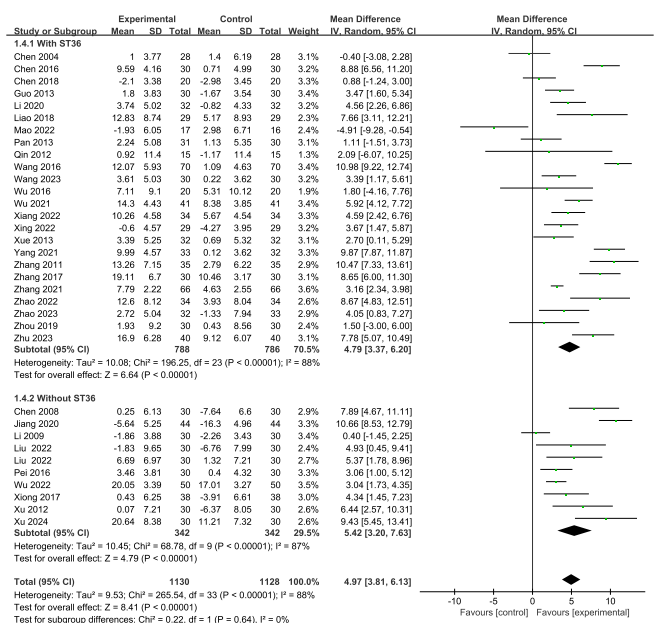


Figure S3 Forest plot for the CD3^+^ level of malignant tumors patients (n=33)

Subgroup analysis was conducted according to acupoint selection of CD3^+^


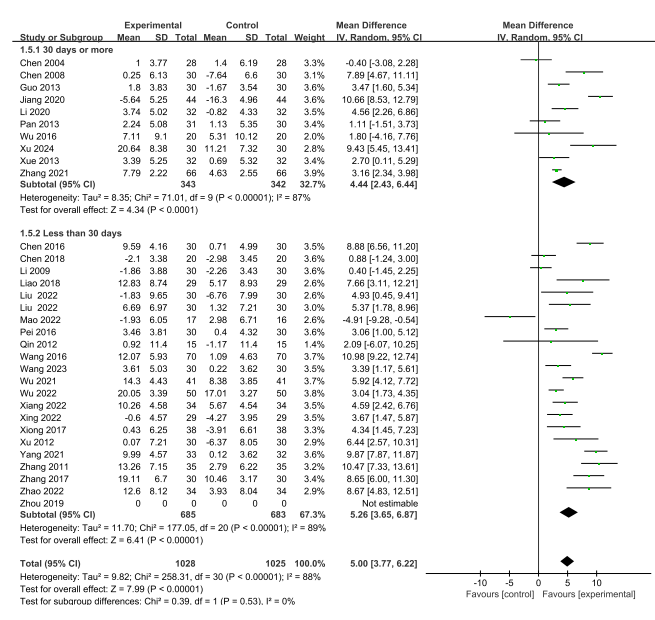


Figure S4 Forest plot for the CD3^+^ level of malignant tumors patients (n=31)

Subgroup analysis was conducted according to duration of treatment of CD3^+^


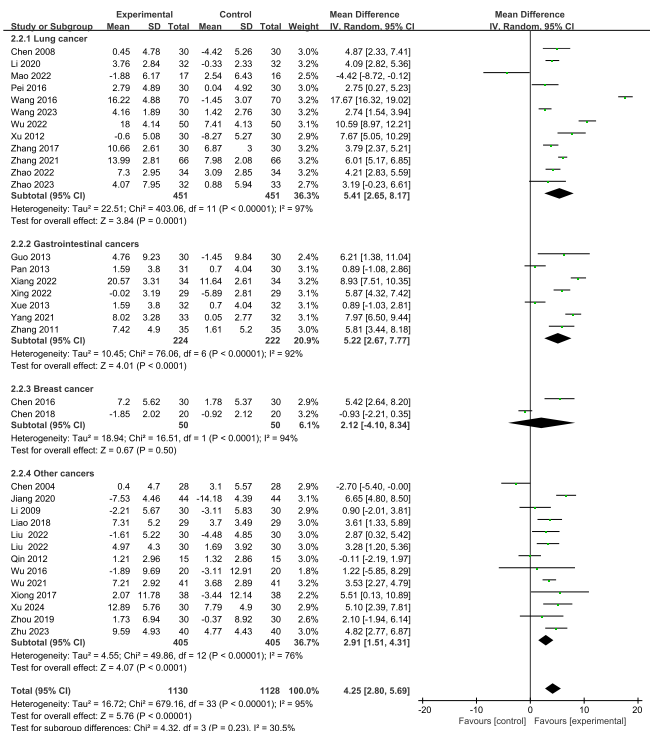


Figure S5 Forest plot for the CD4^+^ level of malignant tumors patients (n=33)

Subgroup analysis was conducted according to cancer typology of CD4^+^


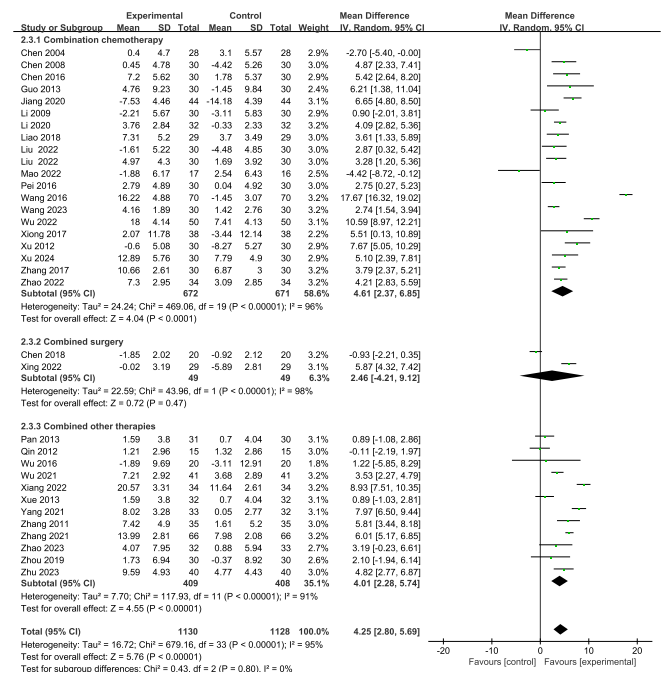


Figure S6 Forest plot for the CD4^+^ level of malignant tumors patients (n=33)

Subgroup analysis was conducted according to clinical intervention of CD4^+^


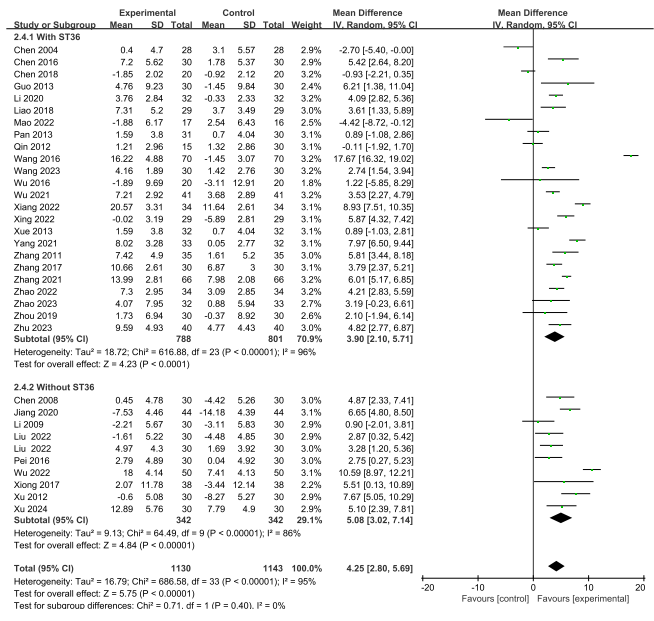


Figure S7 Forest plot for the CD4^+^ level of malignant tumors patients (n=33)

Subgroup analysis was conducted according to acupoint selection of CD4^+^


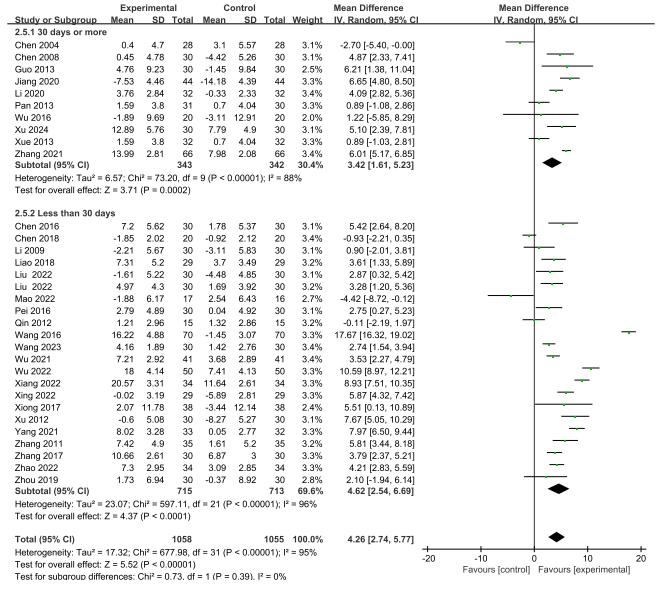


Figure S8 Forest plot for the CD4^+^ level of malignant tumors patients (n=31)

Subgroup analysis was conducted according to duration of treatment of CD4^+^


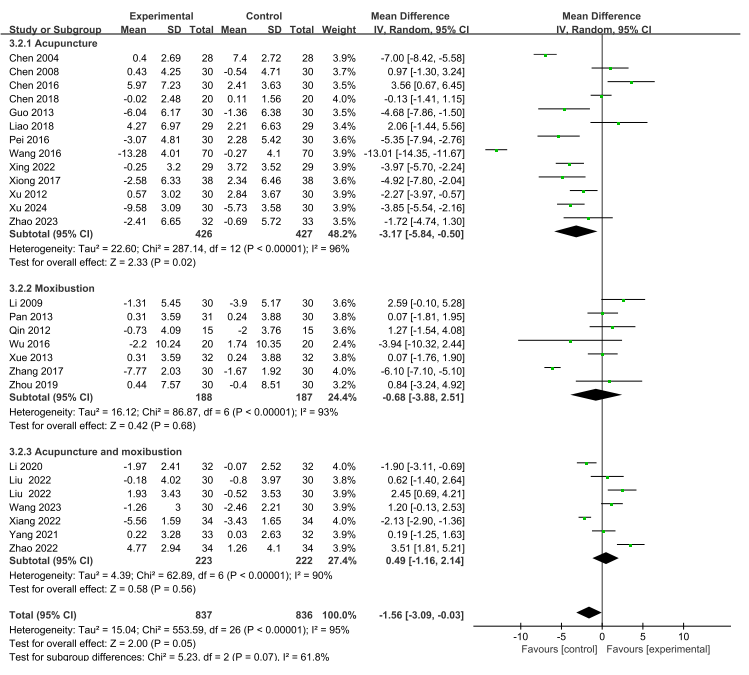


Figure S9 Forest plot for the CD8^+^ level of malignant tumors patients (n=26)

Subgroup analysis was conducted according to intervention group clinical treatment of CD8^+^


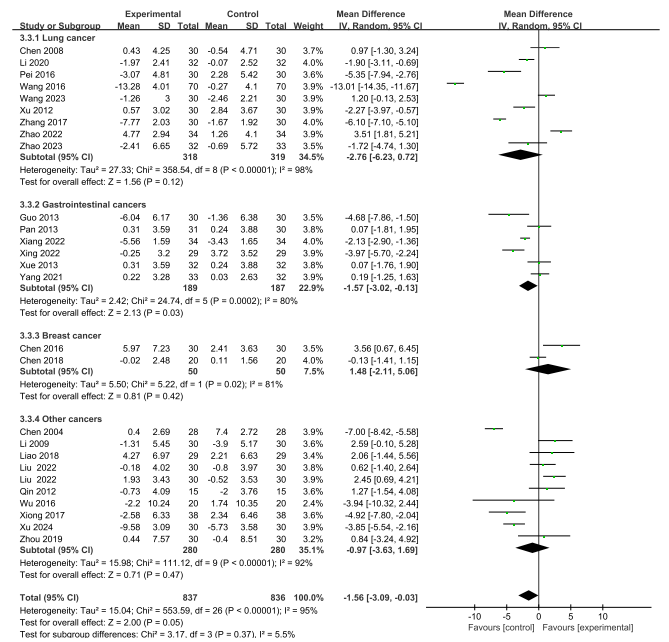


Figure S10 Forest plot for the CD8^+^ level of malignant tumors patients (n=26)

Subgroup analysis was conducted according to cancer typology of CD8^+^
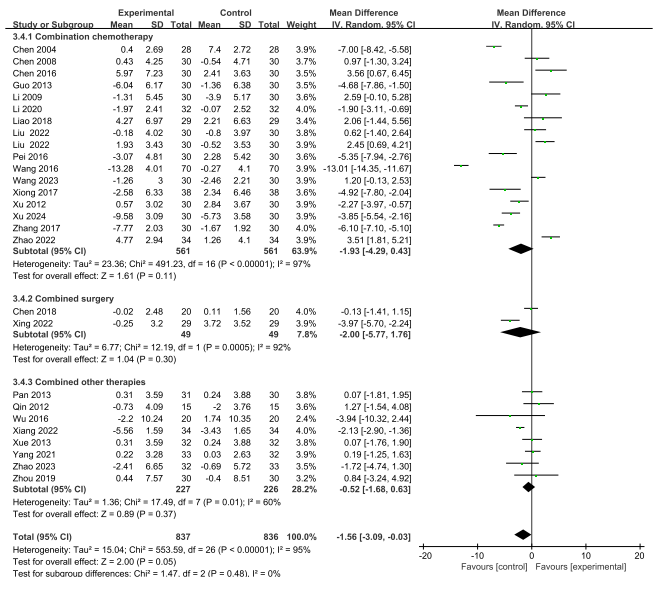


Figure S11 Forest plot for the CD8^+^ level of malignant tumors patients (n=26)

Subgroup analysis was conducted according to two groups clinical intervention of CD8^+^
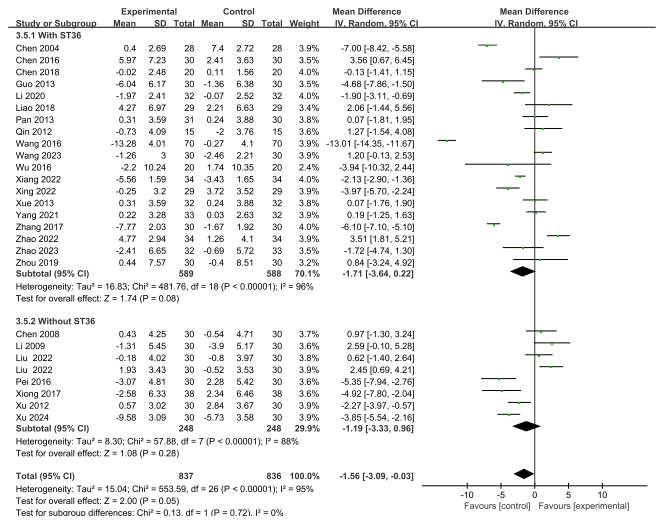


Figure S12 Forest plot for the CD8^+^ level of malignant tumors patients (n=26)

Subgroup analysis was conducted according to acupoint selection of CD8^+^


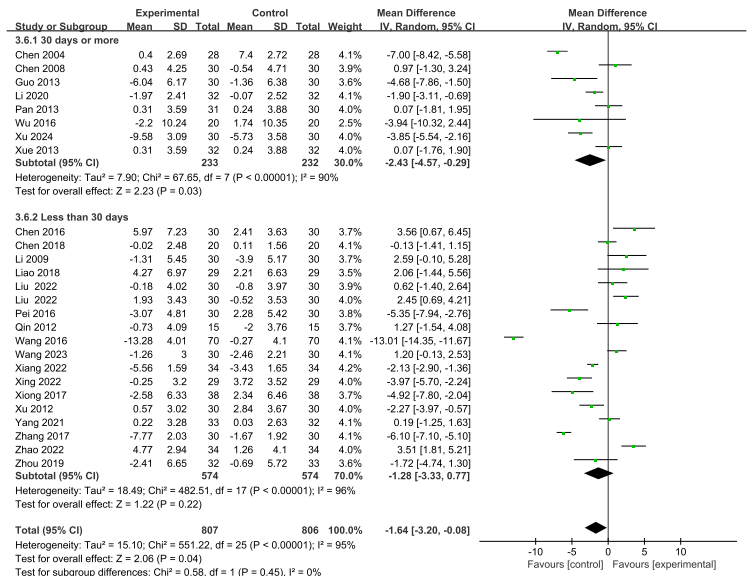
Figure S13 Forest plot for the CD8^+^ level of malignant tumors patients (n=25)

Subgroup analysis was conducted according to duration of treatment of CD8^+^


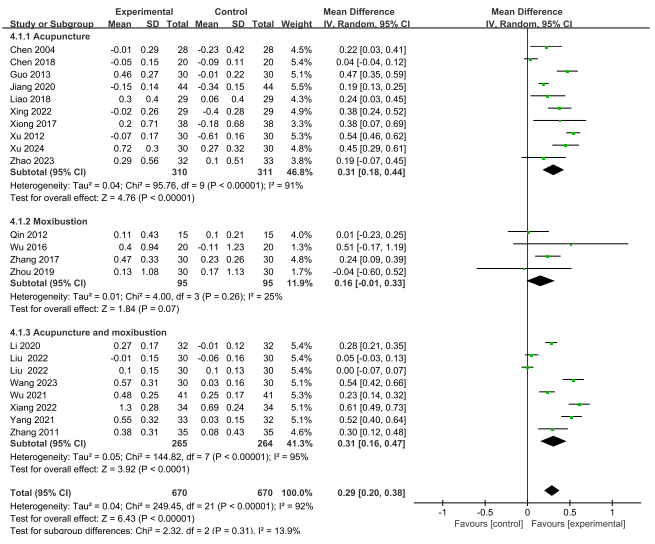


Figure S14 Forest plot for the CD4^+/^CD8^+^ level of malignant tumors patients (n=21)

Subgroup analysis was conducted according to intervention group clinical treatment of CD4^+/^CD8^+^
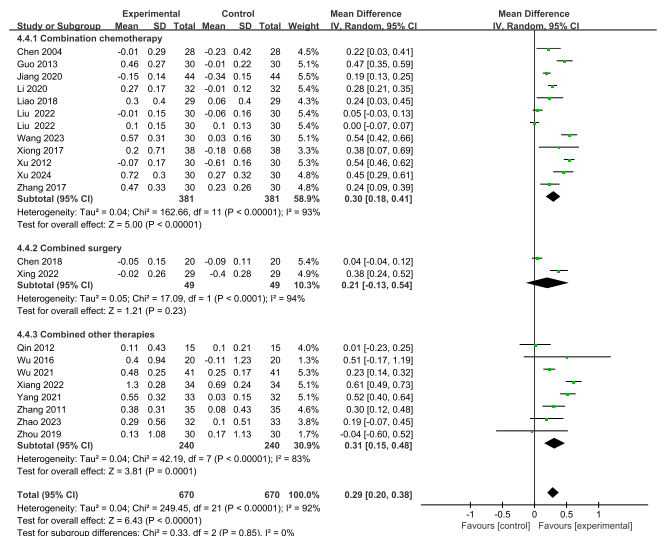


Figure S15 Forest plot for the CD4^+/^CD8^+^ level of malignant tumors patients (n=21)

Subgroup analysis was conducted according to two groups clinical intervention of CD4^+/^CD8^+^


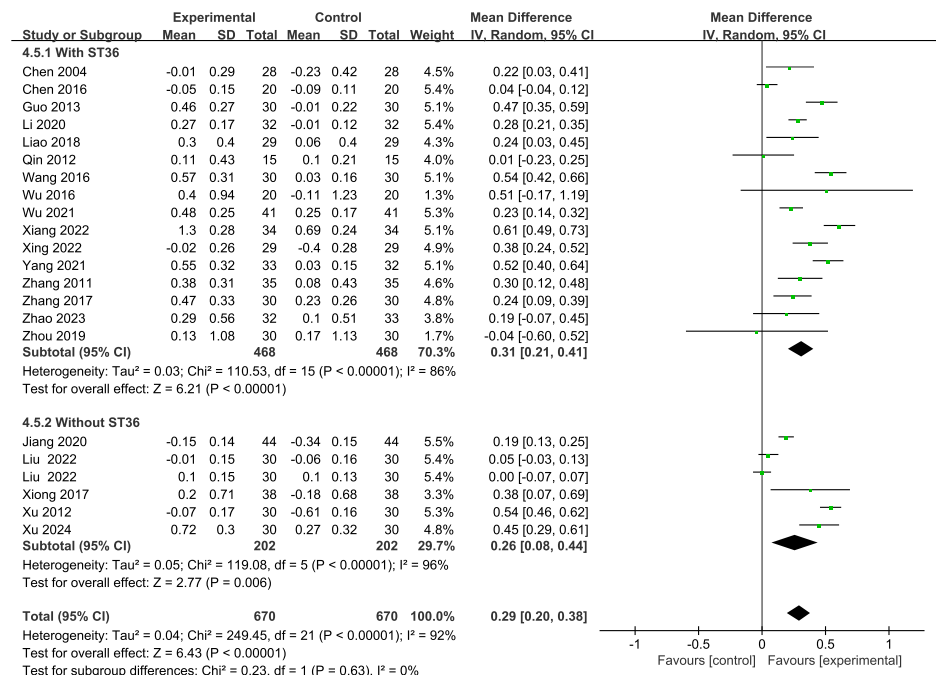
Figure S16 Forest plot for the CD4^+/^CD8^+^ level of malignant tumors patients (n=21)

Subgroup analysis was conducted according to acupoint selection of CD4^+/^CD8^+^


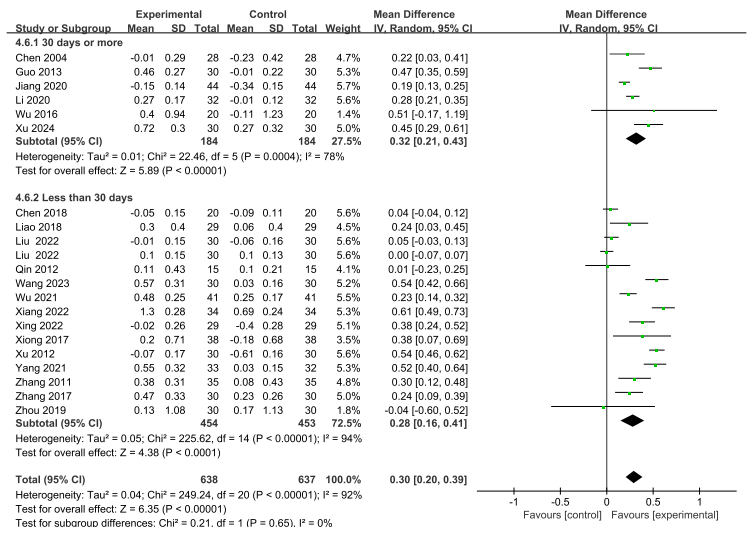


Figure S17 Forest plot for the CD4^+/^CD8^+^ level of malignant tumors patients (n=20)

Subgroup analysis was conducted according to duration of treatment of CD4^+/^CD8^+^


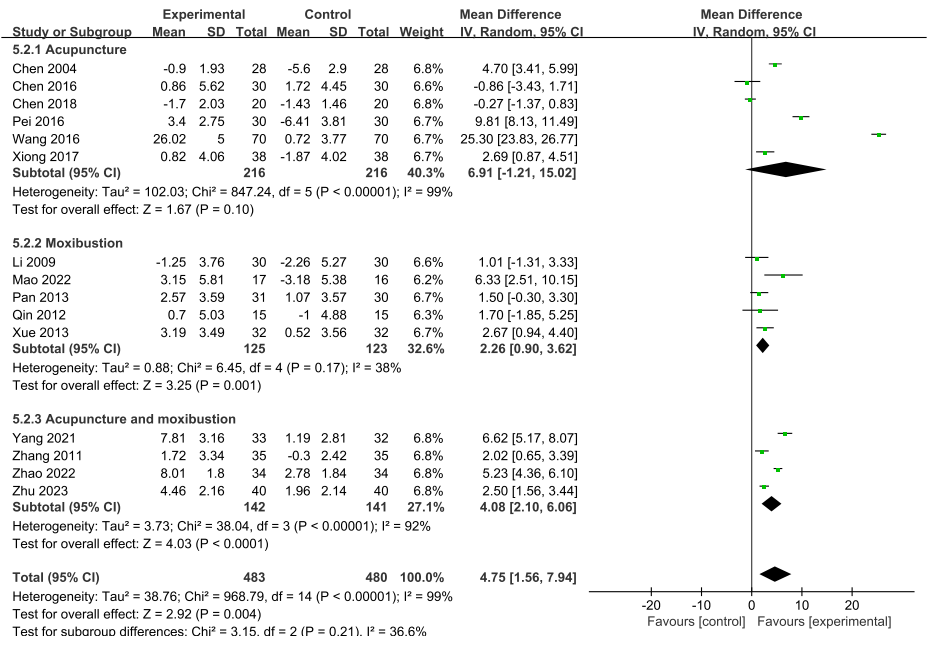
Figure S18 Forest plot for the NK level of malignant tumors patients (n=15)

Subgroup analysis was conducted according to intervention group clinical treatment of NK


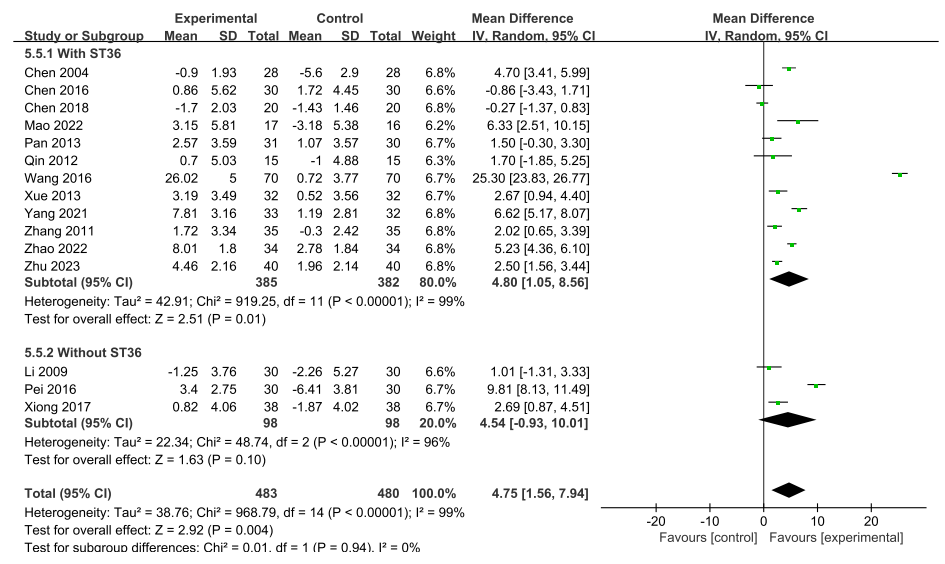
Figure S19 Forest plot for the NK level of malignant tumors patients (n=15)

Subgroup analysis was conducted according to acupoint selection of NK


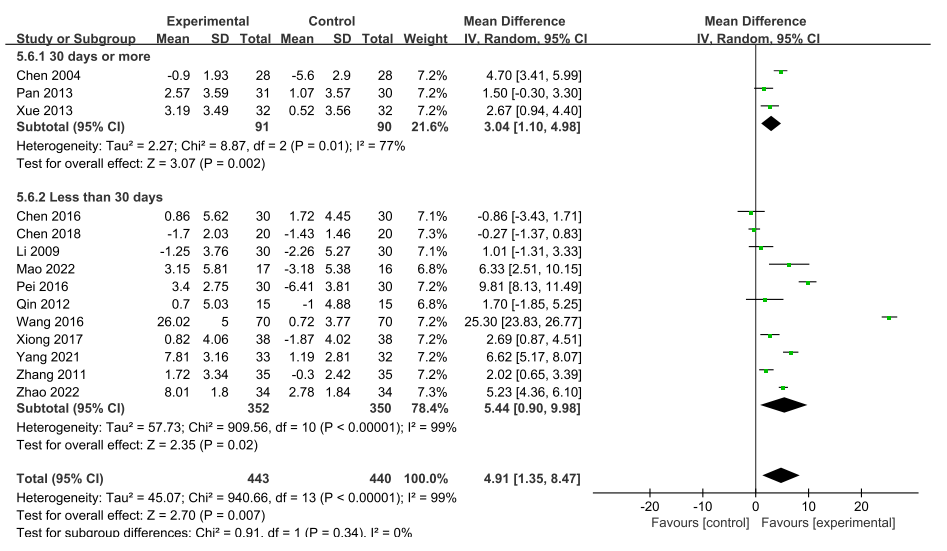


Figure S20 Forest plot for the NK level of malignant tumors patients (n=14)

Subgroup analysis was conducted according to duration of treatment of NK
