# Supplementary material for: Effect of acupuncture and moxibustion on the immune function of patients with malignant tumors: a systematic review and meta-analysis
Source: Front Immunol. 2025 Jul 25;16:1583522. doi: 10.3389/fimmu.2025.1583522 (PMC12331736; doi:10.3389/fimmu.2025.1583522)
Supplement: Supplementary file 2 [file DataSheet2.doc]

**Supplementary File 2.**

**Search strategy used in PubMed**

#1 ( Neoplasms [MeSH Terms])

#2 ((((((((((((((((Tumors[Title/Abstract]) OR (Neoplasia[Title/Abstract])) OR (Neoplasias [Title/Abstract])) OR (Neoplasm[Title/Abstract])) OR (Tumor[Title/Abstract])) OR (Cancer[Title/Abstract])) OR (Cancers[Title/Abstract])) OR (Malignant Neoplasm[Title/Abstract])) OR (Malignancy[Title/Abstract])) OR (Malignancies[Title/Abstract])) OR (Malignant Neoplasms[Title/Abstract])) OR (Neoplasm, Malignant[Title/Abstract])) OR (Neoplasms, Malignant[Title/Abstract])) OR (Benign Neoplasms[Title/Abstract])) OR (Neoplasms, Benign[Title/Abstract]))OR (Neoplasm, Benign[Title/Abstract])) OR (Benign Neoplas[Title/Abstract])

#3 #1 or #2

#4 (Acupuncture Therapy[MeSH Terms]) OR (Electroacupuncture[MeSH Terms])

#5 ((((((((((((Acupuncture Treatment[Title/Abstract]) OR (Acupuncture Treatments[Title/Abstract])) OR (Treatment, Acupuncture[Title/Abstract])) OR (Therapy, Acupuncture[Title/Abstract])) OR (Pharmacoacupuncture Treatment[Title/Abstract])) OR (Treatment, Pharmacoacupuncture[Title/Abstract])) OR (PharmacoacupunctureTherapy[Title/Abstract])) OR (Therapy, Pharmacoacupuncture[Title/Abstract])) OR (Acupotomy[Title/Abstract])) OR (Acupotomies[Title/Abstract])) OR (Electroacupuncture[Title/Abstract])) OR fire needle([Title/Abstract]))

#6 #4 OR #5

#7 T-Lymphocytes[MeSH Terms]

#8 (((((((((((((((((T-Lymphocyte[Title/Abstract]) OR (T Lymphocytes[Title/Abstract])) OR (T cell[Title/Abstract])) OR (Cells, T[Title/Abstract])) OR (Cell, T[Title/Abstract])) OR (T Cells[Title/Abstract])) OR (T Lymphocyte[Title/Abstract])) OR

(Lymphocytes, T[Title/Abstract])) OR (Lymphocyte, T[Title/Abstract])) OR (Thymus-Dependent Lymphocytes[Title/Abstract])) OR (Lymphocytes, Thymus-Dependent[Title/Abstract])) OR Lymphocyte, Thymus-Dependent[Title/Abstract])) OR (Thymus-Dependent Lymphocyte[Title/Abstract])) OR (Thymus Dependent Lymphocytest[Title/Abstract])) OR (T-Cells[Title/Abstract])) OR (T-Cell [Title/Abstract])

#9 #7 OR #8

#10 ((randomized controlled trial[pt] OR controlled clinical trial[pt] OR randomized[tiab] OR placebo[tiab] OR clinical trials as topic[mesh:noexp] OR randomly[tiab] OR trial[ti] NOT (animals[mh] NOT humans [mh])))

#11 #3 AND #6 AND #9 AND #10
